# Exosome Augmentation Technologies for Drug Delivery and Disease Treatment: A Review

**DOI:** 10.34133/bmr.0318

**Published:** 2026-02-19

**Authors:** Jun Wu, Ruibin Li, Lu Cao, Peiqi Wang, Shiqi Jiang, Yan Chen, Haoxin Fu, Xinhao Xu, Guanyang Lin, Lanjie Lei, Ren-ai Xu

**Affiliations:** ^1^ The First Affiliated Hospital of Wenzhou Medical University, Wenzhou, Zhejiang 325000, China.; ^2^ Key Laboratory of Artificial Organs and Computational Medicine in Zhejiang Province, Shulan International Medical College, Institute of Translational Medicine, Zhejiang Shuren University, Hangzhou, Zhejiang 310015, China.

## Abstract

Exosomes are nanovesicles secreted by cells to exchange materials and information. Recent studies have revealed that these modified nanovesicles can be powerful tools for the diagnosis and treatment of diseases. However, few studies have reported on the acquisition and application of these functionalized exosomes. Therefore, this study provides a systematic summary of the entire process of isolation, functionalization, modification, and application of enhanced exosomes and recent progress in this field. First, the process of exosome production and principles of disease treatment are elucidated. Thereafter, the methods of exosome isolation are summarized, with a focus on improved technology centered on aptamer technology and new technology represented by microfluidics. Next, the functional modifications of the exosomes are classified and summarized. Finally, new breakthroughs in the diagnostic and therapeutic capabilities of function-enhancing exosomes compared with those of traditional exosomes are summarized, especially in terms of how these exosomes can be used in bioimaging, photothermal therapy, and other means of achieving a quantum leap in detection and therapeutic efficacy. This paper summarizes the latest research findings on engineered exosomes, with a particular focus on emerging technologies such as microfluidics and aptamers that hold significant potential. It provides a thorough analysis of their respective advantages and limitations, aiming to offer actionable insights for the future advancement and more complex applications of exosomes.

## Introduction

Exosomes are double membrane-structured nanovesicles secreted by cells that carry various biomolecules, including lipids, proteins, and nucleic acids [1]. Initially, exosomes were thought to be waste products of cellular damage and, thus, did not receive much attention; however, in recent years, studies have shown that exosomes have specific functions and that these vesicles are secreted by cells into the body fluid circulation and play an important role in intercellular communication [2,3]. They carry bioactive molecules to proximal and distal cells and help maintain physiological communication involved in internal homeostasis or pathological responses [4]. They are widely distributed in all biological fluids, including blood, urine, saliva, and cerebrospinal fluid. Under physiological conditions, exosomes participate in regulating multiple critical life processes including immune responses, tissue repair, neural signaling, and metabolic balance, serving as indispensable mediators in maintaining the dynamic equilibrium of the body’s functional networks. Their extensive and intricate regulatory functions increasingly highlight their fundamental role in life processes. In recent years, exosomes have attracted considerable interest as diagnostic and therapeutic biomarkers [5,6]. Numerous studies have shown that exosomes are effective in treating inflammation, neurological disorders, cancer, and skin trauma [7–10]. The demand for high-quality exosomes with new functions is constantly increasing.

The purity of exosomes directly affects their use, and a convenient means of separation can influence their prospects for future applications. Therefore, efficient and reliable separation techniques are needed; ultracentrifugation, ultrafiltration, and immunoaffinity capture have been widely used for isolating exosomes [11,12]. However, the exosomes obtained by these classical techniques alone cannot adapt to the needs of the current experimenters, and the use of newer, simpler, and more efficient means such as microfluidics has been emphasized [13]. Simultaneously, the introduction of new concepts such as aptamers has led to an iterative update of classical techniques, and improved techniques based on these new studies have compensated for their shortcomings and are becoming a highly favored means of separation [14].

In the process of utilizing exosomes for diagnosing and treating diseases, the shortcomings of natural exosomes are constantly exposed, and as the research on exosomes continues to deepen, functionally enhanced exosomes obtained by a combination of genetic engineering [15], chemical modification [16], and membrane fusion technology [13] continue to emerge. These novel exosomes have effectively solved a series of practical problems such as biological barriers and immune microenvironment inhibition in the tumor microenvironment (TME) by means of enhanced targeting and promotion of uptake [17,18]. In addition, these exosomes have successfully entered new fields that traditional exosomes are unable to reach by means of artificial modification such as bio-imaging [19] and photothermal therapy (PTT) [20], with great success.

However, there are few systematic and specific summaries of highly performing, functionally enhanced exosomes. This paper reviews various processes of exosome isolation, modification, and application in light of recent research progress. First, we summarize the exosome extraction technology in recent years, focusing on the principle, specific operation process, and other aspects of the microfluidic as a representative of the new separation technology. Although these technologies have shown potential in exosome extraction, previous articles have rarely classified them in detail. Here, we summarize various techniques derived from microfluidics based on the principles of immunoaffinity, electrophoresis, and acoustic force to outline ideas for the development of novel and efficient exosome isolation techniques. Subsequently, the directions of exosome modification are introduced from the perspectives of enhancing delivery ability and therapeutic effect, and the advantages and shortcomings of various modification methods are explained. The idea that it could be used as a source of membrane fusion or therapeutic content through the development of novel exosomal feedstocks, particularly through the discovery of new natural exosomes with therapeutic capabilities, is also discussed. Finally, we outline the application of various function-enhancing exosomes in recent years, summarizing new breakthroughs of the novel exosomes in overcoming biological barriers, their use in the fields of immunotherapy and medical imaging, and combining them with therapeutic strategies, such as photodynamic therapy (PDT), PTT, and ultrasound-targeted disruption. We also highlight the problems faced currently and present prospects for the development of exosomes as functional enhancements for the diagnosis and treatment of diseases.

## Process of Exosome Genesis

Exosomes are primarily generated through 2 plasma membrane invaginations [21]. The first plasma membrane invagination leads to the formation of an early-sorting endosome, which continues to mature into a late-sorting endosome. Subsequently, a multivesicular body (MVB) is formed, at which point a second plasma membrane invagination occurs that can either fuse with lysosomes and autophagosomes for degradation or with the plasma membrane to release intraluminal vesicles (ILVs). The released ILVs are exosomes (Fig. S1).

Exosome biogenesis can be categorized into endosomal sorting complexes required for transport (ESCRT)-dependent and non-ESCRT-dependent strategies. ESCRT is a protein complex that binds MVB membranes and consists of approximately 30 proteins that are assembled via associated proteins (VPS4, VTA1, and ALIX) [22]. ESCRT is divided into 4 types, ESCRT-0, -I, -II, and -III, of which ESCRT-0, -I, and -II are involved in content sorting, and ESCRT-III is involved in membrane deformation and fission. ESCRT-0 interacts with TSG101 to recruit ESCRT-I [23]. Subsequent aggregation of ESCRT-I and ESCRT-II at the neck of the sorter bud promotes the movement of the membrane around the ubiquitinated protein cluster into the bud, confining the contents within the bud. The tension generated by the helix drives the fusion of the membranes at the neck of the mature bud. ILV, thus, enters the lumen of the MVB. In this process, ALIX also interacts with the ESCRT-III protein CHMP4B [24], which forms a multimeric helix in the neck of the developing ILV. The ATPase activity of VPS4A subsequently induces a conformational change in the CHMP4B helix, leading to the breakage of ILV from the restriction membrane of MVBs [25,26]. The ESCRT-independent pathway proceeds through (a) ceramide activation via neutral sphingomyelinase 2 (nSMase2) [27], (b) phosphatidic acid activation via phospholipase D2, or (c) rab31-flotilin-dependent lipid raft formation [28]. Under iron loading, intracellular ferritin promotes exosome release by up-regulating CD63 expression [29]. mTORC1 target activation regulates exocytosis by affecting CD63 expression [30]. In addition, raft proteins in lipid raft structures can bind to shell proteins that form outgrowth vesicles and affect vesicle formation and secretion [31].

**Fig. 1.**
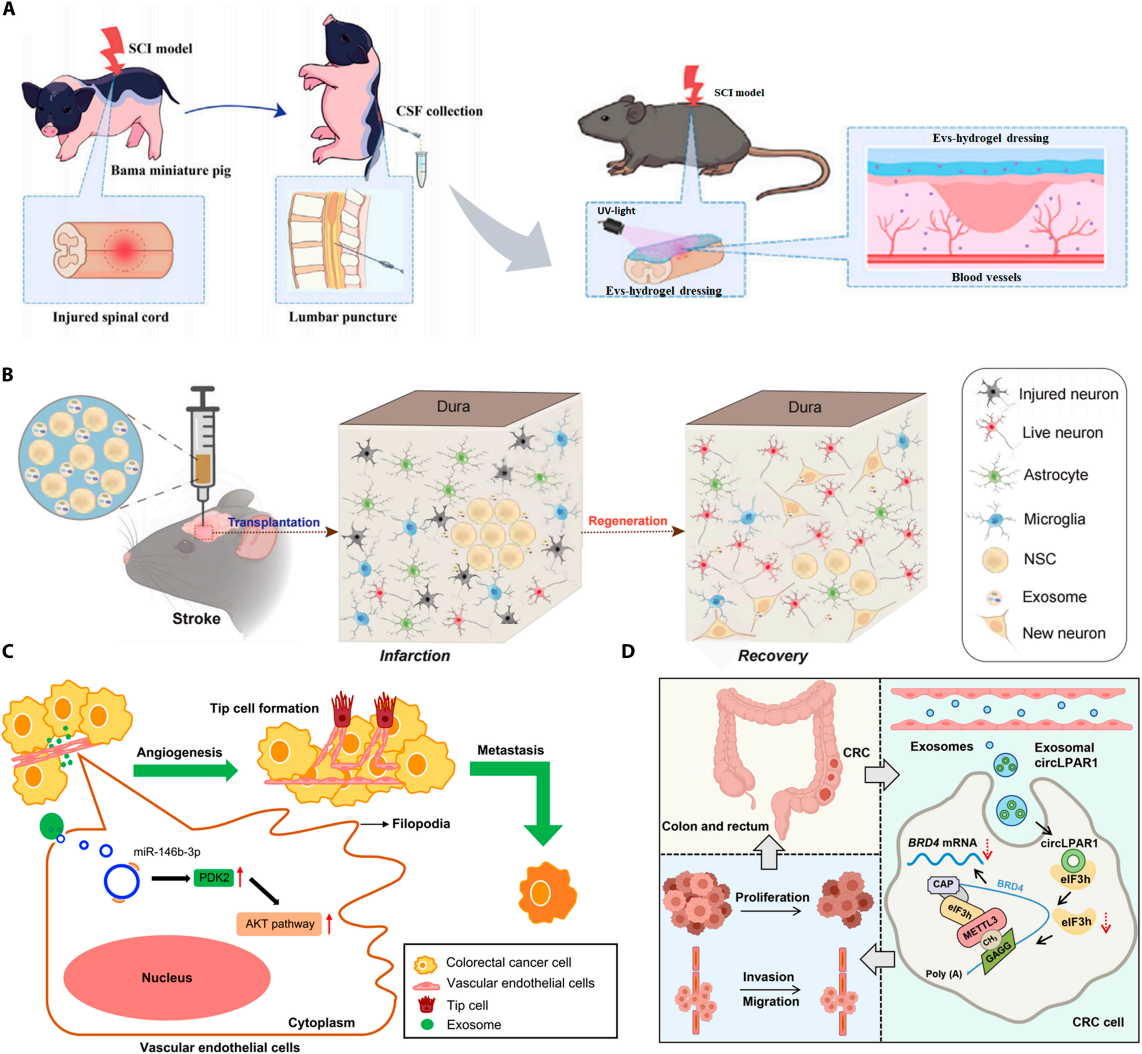
Roles played by exosomes under physiological conditions. (A) Extracellular vesicles derived from cerebrospinal fluid after porcine spinal cord injury can promote vascular regeneration in mice through specific signaling pathways. Reproduced with permission [38]. Copyright 2023, Elsevier Ltd. (B) Neural stem cell-derived exosomes enhance the therapeutic effect of neural stem cell transplantation on cerebral ischemia in mice. Reproduced under terms of the CC-BY license [39]. Copyright 2023, eLife. (C) Colorectal cancer cell-derived exosomes can promote angiogenesis and colorectal cancer metastasis by activating the Akt signaling pathway under physiological conditions. Reproduced under terms of the CC-BY license [40]. Copyright 2023, Springer Nature. (D) Exosomes produced by some cancer cells are regarded as cancer markers. Reproduced under terms of the CC-BY license [41]. Copyright 2022, Springer Nature.

Different MVBs travel to different destinations. They may fuse with lysosomes, leading to degradation of the endocytic vesicle structure and contents [32], or with the plasma membrane of the mother cell, leading to the release of endocytic vesicles into the extracellular space by cytokinesis [33]. The released exosomes play an important role in regulating the behavior of neighboring or distant cells. Since exosomes are a substance produced by the cells themselves, their unique immunogenicity has attracted much attention from the scientific community owing to their immunotherapeutic potential, either auto- or allogeneic. Thus, exosomes are also recognized as promising nano-drug delivery routes for a wide range of biomedical applications. They have shown marked regenerative effects in models of myocardial infarction and renal, hepatic, and neurological injury by reducing inflammation and apoptosis while promoting proliferation and angiogenesis. Anti-iron death exosomes from adipose-derived mesenchymal stem cell (ADSC) intranasal administration have been used in palliative treatment of ischemic brain injury [34]. The exosome miR-27b-3p, a mesenchymal stem cell-derived exosome, attenuates carbon tetrachloride-induced liver fibrosis [35]. The bone marrow mesenchymal stem cell (BM-MSC)-derived exosome miR-21a-5p alleviates renal fibrosis by targeting PFKM to attenuate glycolysis [36]. The exosome miR-29b-3p released by BM-MSCs can regulate insulin resistance associated with aging [37]. Li et al. [38] obtained extracellular vesicles (EVs) derived from cerebrospinal fluid post-injury by disrupting the spinal cord and successfully demonstrated their pro-angiogenic effects. Zhang et al. utilized NSCs derived from human induced pluripotent stem cells along with exosomes extracted from these NSCs. Subsequent experiments successfully demonstrated that these neurostem cell-derived exosomes enhanced the therapeutic efficacy of neurostem cell transplantation for mouse cerebral ischemia [39]. In contrast, Chen et al.’s research highlights the information regulatory role of exosomes. Their team discovered that exosomes derived from colorectal cancer (CRC) cells promote angiogenesis and CRC metastasis by activating the Akt signaling pathway under physiological conditions [40]. Zheng et al. [41] further identified the plasma-derived exosome circLPAR1 as a promising diagnostic biomarker for CRC. These findings collectively demonstrate that exosomes and their secreted factors play important roles in both tissue regeneration and information regulation, indicating that exosomes serve as crucial signaling tools under physiological conditions (Fig. 1).

## The Mechanism and Fundamental Applications of Exosome Therapy in Disease Treatment

### Treatment of inflammation

Inflammation is a direct, nonspecific response to invading organisms, foreign bodies, necrotic cells, irritants, and tumor cells. This innate immune response leads to typical manifestations, such as redness, swelling, pain, and fever [42]. Exosomes influence various inflammatory processes [43]. Exosomal contents contain nucleic acid components, namely, DNA and miRNA, that direct and regulate the innate and adaptive immune responses of the body and modulate the immune response by influencing gene expression and signaling pathways in recipient cells. Exosomal miRNAs can influence dendritic cell (DC) maturation by exchanging with each other and suppressing gene expression [44]. In previous studies, miR-1249-3p in natural killer cell-derived exosomes significantly induced cellular insulin sensitivity and alleviated inflammation [45], and exosomes loaded with DNA from intracellular bacteria were able to stimulate the cGAS-STING signaling of nearby cells, effectively activating the innate immune response [46,47]. Therefore, the role of nucleic acid content of exosomes in antigen presentation should not be overlooked. During activation of the immunomodulatory system, macrophages produce large amounts of exosomes containing interferon (IFN)a and IFNg, tumor necrosis factor alpha, and interleukin (IL)-containing exosomes for promoting DC maturation and CD4^+^ and CD8^+^ T cell activation, and the body can enhance bacterial and antigen presentation by enhancing macrophage-derived exosomes [48].

Exosomes can transport various inflammatory factors. By targeting and delivering IL-10-containing exosomes to macrophages, they can effectively inhibit the inflammatory response, thus alleviating atherosclerosis [49]. MSC-derived exosomes (MSC-Exo) can attenuate neurological inflammation by inhibiting Nrf2/NF-κB/NLRP3 signaling [50]. In one study, exosomes derived from MSC-L alleviated dextran sodium sulfate-induced acute colitis in C57 and IL-10 mice by increasing anti-inflammatory cytokine (IL-10) levels [51].

### Wound repair

**Fig. 2.**
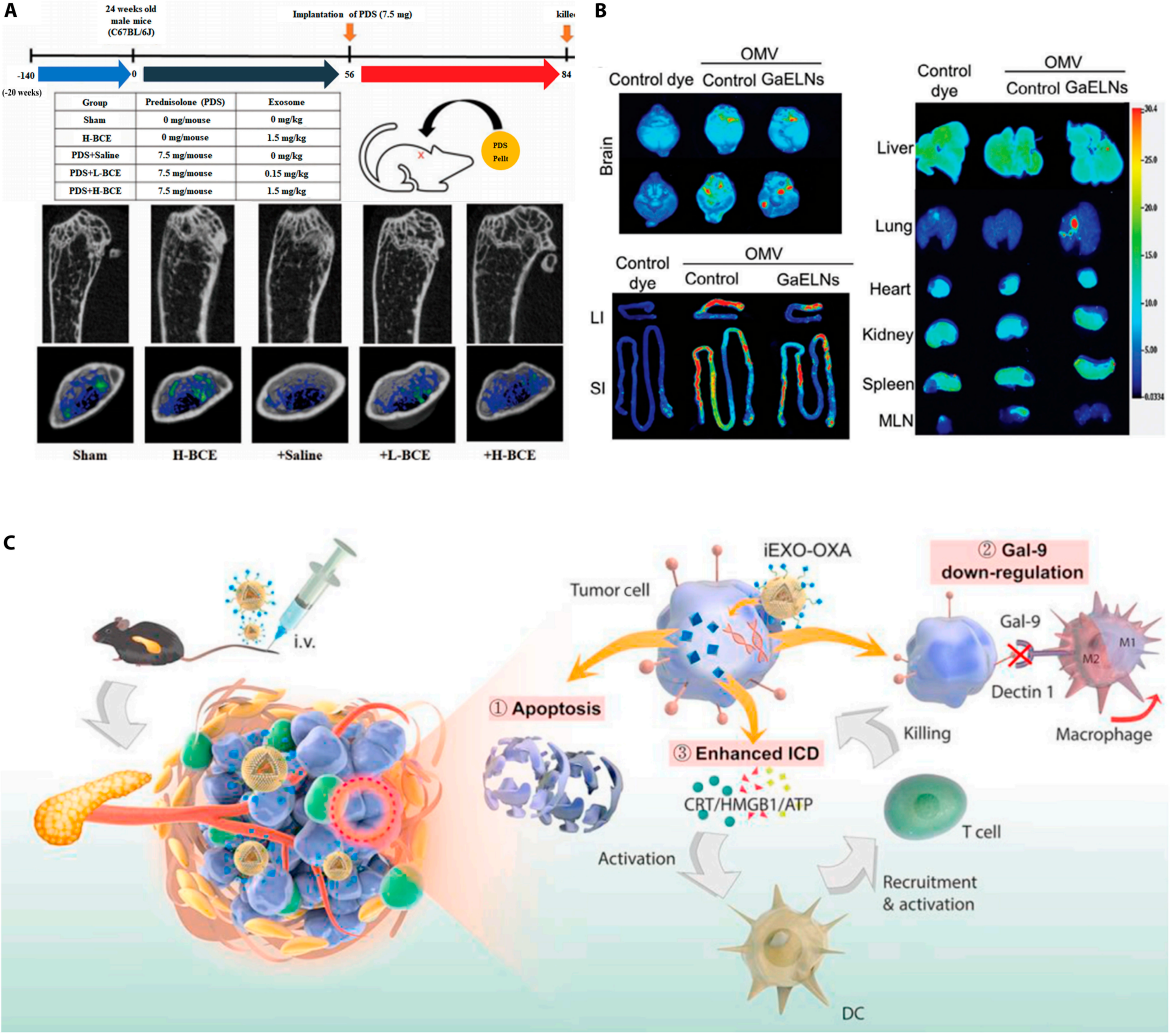
Exosome applications. (A) Exosomes derived from cow’s milk improve bone health in osteoporotic model mice. Reproduced with permission [57]. Copyright 2020, Elsevier Ltd. (B) Exosomes can reverse type II diabetes by training gut bacteria through the gut–brain axis. Reproduced under terms of the CC-BY license [70]. Copyright 2024, Wiley-VCH. (C) Modified exosomes can support tumor immunotherapy by altering the tumor microenvironment. Reproduced with permission [84]. Copyright 2021, Elsevier Ltd.

Several exosomes favor post-traumatic repair of the skin, bone, and various organs [52–57] (Fig. 2A). Among these, exosomes, secreted directly or modified by adipose-derived stem cells (ADSCs), and MSCs have been widely used for skin wound repair because of their excellent properties [58,59]. In one study, hypoxia-treated ADSC-derived exosomes enhanced wound healing in diabetic mice by delivering circ-Snhg11 and inducing M2-like macrophage polarization [60]. MSC-Exos have been found to promote wound healing by influencing glucose, lipid, and amino acid metabolism to regulate wound healing and scar shape [61]. Hu et al. [62] isolated exosomes from the supernatant of pioglitazone-treated MSCs (PGZ-Exos) and found that PGZ-Exos stimulated angiogenesis and accelerated diabetic wound healing by enhancing the viability of human umbilical vein vascular endothelial cells following high glucose injury. In addition, exosomes can promote bone tissue regeneration by stimulating the proliferation, differentiation, and mineralization of osteoblasts as well as by increasing the abundance of bone differentiation markers (osteopontin, alkaline phosphatase, and collagen type I). Hwang et al. [63] found that nanovesicles extracted from yam promote longitudinal bone growth and mineral density in ovariectomized osteoporotic mice. Lei et al. [64] extracted exosomes secreted by periodontal ligament stem cells for treating periodontitis in rats, and they found that exosomes enhanced the osteogenic capacity of endogenous stem cells under an inflammatory milieu and promoted alveolar bone regeneration.

Exosomes are effective in repairing lung, kidney, heart, and other organ injuries [65]. Fibroblast reticulocyte-derived exosomes promote kinase PINK1-dependent mitochondrial autophagy and inhibit lipopolysaccharide (LPS)-stimulated activation of NLRP3 inflammatory vesicles in primary renal tubular cells, thereby protecting renal function in C57BL/6 mice after cecal ligation and puncture-induced sepsis [66]. Using a C57BL/6 mouse model of unilateral ureteral obstruction and cisplatin-stimulated injury of the epithelial cells of HK-2 cells, Yu et al. [67] demonstrated that human umbilical cord MSC-Exos could regulate necroptosis through miR-874-3p, attenuate renal tubular epithelial cell injury, and promote the repair of the affected area in vitro and in vivo. Cao et al. [68] imaged the biodistribution of MSC-Exos in ischemia/reperfusion-induced acute kidney injury in mice using the spectral in vivo imaging system and demonstrated that MSC-Exos mitigated cell cycle arrest and apoptosis in renal tubular epithelial cells via miR-125b-5p/p53, attenuated ischemic acute kidney injury, and mitigated tubular injury in a dose-dependent manner in mice. Shen et al. [69] successfully isolated ADSC-Exos and demonstrated that miR-125b-5p alleviates sepsis-induced, inflammation-induced iron death in pulmonary microvascular endothelial cells by regulating Keap1/Nrf2/GPX4 expression, thereby ameliorating acute lung injury in patients with sepsis. Sundaram et al. took a novel approach by training mouse gut microbiota using exosome-like secretions from garlic. The outer membrane vesicles secreted by the trained microbiota were subsequently taken up by brain microglia, thereby suppressing high-fat diet-induced brain inflammation. Follow-up studies revealed that the signaling pathways involved in this process also interact with insulin signaling, partially reversing type 2 diabetes [70] (Fig. 2B).

Exosomes exert tangible therapeutic effects on the healing of various wounds in vivo and in vitro by promoting cell growth, controlling the release of inflammatory factors, and inhibiting cell cycle abnormalities and apoptosis.

### Cancer diagnosis and treatment

#### Cancer diagnosis

Exosomes are formed through pathways by which cells deliver intracellular substances over long distances, and they are widely distributed and stable in biological fluids. Cancerous cells undergo a series of changes in the types and proportions of their contents, including nucleic acids, proteins, lipids, sugar structures, and metabolites of the mother cell, which in the past have been considered important indicators for the diagnosis of cancerous cells. In addition, molecules carried to the surface of exosome membranes reveal the origin of these vesicles, allowing the classification of vesicle types and enrichment of features from tissue-specific sources [71,72]. Therefore, many researchers have considered exosomes to be an effective tool for diagnosing cancer. Zheng et al. [41] demonstrated that the plasma exosome circLPAR1 is a promising diagnostic predictor of CRC using circRNA pull-down, proteomic analysis, and RNA immunoprecipitation assay and described its role in biological regulation of colorectal tumorigenesis. Zhou et al. [73] collected 202 independent plasma samples and validated them by real-time quantitative reverse transcription polymerase chain reaction (PCR) in 32 pairs of endometrial tumors and adjacent normal tissues and by droplet digital PCR in matched plasma samples from 12 patients preoperatively and postoperatively; they found that plasma-derived exosome miR-15a-5p was an effective diagnostic biomarker for early cancer detection. Chang et al. [74] collected data of 110 patients with non-small cell lung cancer and found that the expression levels of plasma versican and plasma exosomal versican were significantly up-regulated in patients with non-small cell lung cancer and significantly elevated in patients with advanced-stage cancer compared to in those with early-stage cancer, proving that the high expression of plasma exosomal versican could be used as a predictor of non-small cell lung cancer risk. Enriched proteins such as EGFR, GRB2, and SRC found in exosomes secreted by tumor cells are associated with cancer metastasis and progression and are considered to be some of the potential markers that can be used for early cancer detection [75–78].

#### Cancer treatment

Cancer cells, as exosome releasers, can also act as exosome receivers, so that drug-loaded exosomes can be targeted, delivered, and cytophagocytosed into tumor cells for therapeutic purposes. Tian et al. [79] reported that modified mouse immature DCs expressed a well-characterized exosomal membrane protein (Lamp2B) fused to the l-charactin-specific internalizing RGD (iRGD) peptide (CRGDKGPDC) and successfully delivered adriamycin-loaded exosomes to the tumor tissues in subsequent animal experiments. Saari et al. [80] used differential centrifugation to isolate exosomes from LNCaP and PC-3 prostate cancer cell cultures loaded with paclitaxel and showed that cancer cell-derived exosomes can carry the drug into the cells via the endocytic pathway and increase their cytotoxicity.

Drug resistance is also a reason behind the difficulty in fully curing cancer, and in addition to circumventing it by means of membrane fusion, exosomes can be utilized to reverse drug resistance in cancer cells. Yu et al. [81] successfully demonstrated that the exosome LOC85009 inhibited docetaxel resistance by regulating ATG5-induced autophagy through the USP5/USF1 axis, suggesting that LOC85009 can be a target for drug resistance reversal in the treatment of lung adenocarcinoma. Liang et al. [82] utilized engineered exosomes to simultaneously deliver the anticancer drug 5-FU and the miR-21 inhibitor oligonucleotide (miR-21i) to Her2-expressing cancer cells, extracted the exosomes produced by the cells, and subsequently introduced purified engineered exosomes into the 5-FU-resistant CRC cell line HCT-1165FR. The results showed that the co-delivery of miR-21i and 5-FU with engineered exosomes effectively reversed drug resistance and significantly enhanced cytotoxicity in 5-FU-resistant colon cancer cells compared with monotherapy with miR-21i or 5-FU.

Various exosomes derived from tumor cells and immune cells, which are useful for cancer treatment and control, exhibit unique compositional characteristics and can be directly involved in anticancer immunotherapy by modulating immune function, and the idea of using exosomes as a cancer vaccine has also emerged [83]. Zhou et al. [84] adopted an immunological approach, successfully reversing the tumor immune microenvironment using drug-loaded bone marrow mesenchymal stem cell exosomes to achieve therapeutic outcomes (Fig. 2C). In a study by Huang et al. [85], the immunogenic cell death inducers, human neutrophil elastase (ELANE) and hirudinol (TLR3 agonist), were loaded into the treatment and co-engineered breast cancer-derived exosomes, resulting in an in situ DC vaccine (HELA-Exos). The targeting, killing, and immune activation effects of the vaccine were subsequently evaluated in vitro, demonstrating that the exosome-formulated vaccine produced effective tumor suppression in a triple-negative breast cancer mouse xenograft model and in patient-derived tumor-like organs. Meng et al. [86] inhibited the growth of tumors in a mouse model of metastatic lung cancer using a vaccine containing exosomes isolated from ES-D3 cells that stably expressed GM-CSF (ES-Exo/GM-CSF); importantly, control exosomes without GM-CSF failed to provide protection against metastatic lung tumors in this experiment. In another study, an iPSC exosome (DC + EXO)-pulsed DC vaccine was obtained by incubating pluripotent stem cell-derived exosomes with DCs for pulsing. Splenic T cells extracted after vaccination effectively killed various tumor cells in vitro, and an activated long-term T cell response prevented melanoma recurrence [87].

Through targeted delivery, reversal of drug resistance, and regulation of immune function, exosomes can play a role in enhancing drug efficacy and long-term tumor prevention, providing a foundation and reference direction for the subsequent development of exosomes and enhancement of technology.

## Exosome Extraction

The above sections illustrate the potential of exosomes in the diagnosis and treatment of various diseases. However, exosomes, as intercellular messengers, are found in body fluids at low concentrations [88]. In addition, the size and similarity between exosomes and other EVs, including other exosomes and microvesicles [89], pose a considerable challenge for the separation technique. Therefore, exosomes are difficult to extract and purify, making their wider application difficult to some extent. Effective separation methods are the basis for enhancing exosomes to be put into use. However, since the mid-1990s, the role of exosomes in intercellular communication has been gradually explored and their extraction and isolation techniques have been iterated, and after decades of data accumulation, a variety of mature and effective isolation methods are now available. In this section, we briefly describe the principles, steps, and advantages and disadvantages of the most widely used extraction techniques.

### Density-difference-based separation techniques

Ultracentrifugation is the gold standard for exosome extraction because of its powerful processing capabilities [1]. In this process, a given mixture is subjected to centrifugal force, resulting in different molecules being layered according to their density. Typically, ultracentrifugation can generate centrifugal forces of up to 100,000 to 150,000 [90] (Fig. S2A). According to their particles including bacteria, viruses, and organelles from samples. In 1989, Johnstone et al. first reported the isolation of exosomes from reticulocyte tissue culture media [91]. Prior to this, Linderstorm-Lang found that after centrifugation in a density gradient tube, objects of a specific density would be suspended in a medium of similar density. Inspired by this finding, in 2013, Webber and Clayton [92] improved the centrifugation method in their study by centrifuging the samples using a sucrose solution, ultimately resulting in purer and higher-purity samples; however, this method is still considered the simplest and most effective method for exosome extraction. The following is a brief description of the routine ultracentrifugation procedure: first, depending on the cleanliness of the sample and its origin, large biological particles and live cells are removed by centrifugation at low speeds (often 300 *g*), and several centrifugation cycles are then performed at different speeds starting from 2,000 *g*. Finally, during ultracentrifugation at 100,000 *g*, buffer is added to reduce the quantity of contaminating proteins for further purification of exosomes. This method has been widely used for a variety of samples such as plasma [93], milk [94], saliva [90], cerebrospinal fluid [38], and microorganisms [95]. This method can be applied to large-scale exosome preparation using liquid concentration [96]. However, this method still has drawbacks. In addition to requiring a large amount of time for centrifugation as well as equipment and operators, the high shear force generated during multiple high-speed centrifugations causes damage to the vesicles, resulting in the deterioration of exosome quality, affecting their application. Exosomes extracted by the ultra-isolation method undergo substantial deformation and aggregation under centrifugal forces of up to 10,000 *g* [97,98], posing a potential risk to the correctness of the conclusions of subsequent experiments with exosomes produced by this method. In addition, highly viscous biofluids require longer ultracentrifugation steps and higher centrifugation cycles, which may compromise exosome integrity. The phenomenon that target exosomes can bind to sucrose, iohexanol, or iododiglycol used in density gradient centrifugation should not be overlooked. In general, however, ultracentrifugation is the most efficient and cost-effective method for extracting exosomes.

### Affinity capture-based separation techniques

#### Immunoaffinity capture techniques

A large number of molecules specific to mother cells, such as CD9, CD63, CD81, CD82, Hsp70, ALIX, and Ep-CAM, are present on the surface of exosomes generated by the division of cellular membrane depressions [99]. Long before the discovery of exosomes, a well-established technology based on specific molecules was being used to separate specific components: immunoaffinity capture technology. Since this technique does not rely on force separation, it does not cause mechanical damage to the product, retains its integrity, and also has strong specificity, which are precisely the characteristics required for exosome extraction. In this technique, antibodies specific to exosome surface proteins are immobilized on a substrate, followed by an elution step to separate the two and collect the target product [100] (Fig. S2B). Magnetic beads are the most commonly used immobilization substrates, and after the antibody immobilizes on their surface and captures the exosome, the magnetic properties of the beads can be used to separate the system together from the mixed solution. In addition to the commonly used magnetic beads, microchips are popular substrates for developing immunoaffinity-based exosome isolation systems. Liu et al. reported the efficacy of a total exosome isolation chip called ExoTIC. This method can also be translated into a clinical diagnostic method that can be used to directly bind and detect disease-specific markers such as EpCAM, CD133, and EGFR on exosome surfaces [101]. In a recent study, Wang et al. [102] achieved selective isolation of multiple simultaneous exosome subpopulations and conducted subsequent proteomic analysis using an integrated microfluidic platform of Sub-ExoProfile chips immobilized with 3 specific exosome capture antibodies (CD81, EpCAM, and HER2), demonstrating the feasibility of this technology in capture-analysis integration.

The main problem of this technique is related to the disassembly of the exosome after binding to the immobilized matrix, which is prone to decreased yield, inactivation of the product, or even rupture, if the exosome cannot be eluted smoothly. To compensate for this shortcoming, Zhang et al. successfully developed a novel immunoaffinity sheet material in which anti-Tim4 antibody was bound to an organometallic framework. The addition of a chelating agent under neutral conditions allowed this novel material to release captured exosomes easily, and this treatment technique required mild conditions and a high release rate. Exosome inactivation was avoided [103]. In addition, most of the antibodies used in this method are not specific enough for yielding high-purity exosomes, leading to impurities with similar bound proteins. Therefore, the discovery and application of more specific antibodies is an endeavor of this method, and in addition to proteins, other landmarks on the membrane surface, such as glycoproteins, lipids, and polysaccharides, can also be considered markers of immunocapture [104,105].

Overall, the immunoaffinity capture technique is an effective and unique separation method that is not based on the physical properties of the target product, but its high cost limits its large-scale widespread application. In practice, it is more suitable for use in combination with other methods such as ultrafiltration.

#### Aptamer technology

An aptamer, also known as a chemical antibody, is a short single-stranded DNA or RNA sequence. As the name suggests, it has the advantage of being chemically synthesized in vitro and highly specific for binding [106]. Aptamers have a complex and sophisticated tertiary structure that is susceptible to factors such as temperature, pH, and solution environment, thus changing the strength of antigen binding, allowing us to easily capture and release exosomes [107]. However, this poses a considerable challenge to the synthesis and design. To screen and design aptamers more efficiently, the Systematic Evolution of Ligands by Exponential Enrichment method is commonly used to select aptamers from a random pool using an iterative screening procedure based on their affinity for target molecules (e.g., proteins, small molecules, or even cells) [108]. As research on aptamers continues to advance, researchers have established a database (https://www.aptagen.com/apta-index) based on the sequence information of a large number of different aptamers, and specific biomarkers can be searched through the database, thus facilitating screening and synthesis by researchers. Currently, as an emerging technology, aptamer capture technology has not yet been widely used, but its low batch-to-batch variation, controlled capture and release capability, and strong specific capture capability indicate its potential for being developed in the future and successfully used in a variety of applications.

#### Other affinity capture methods

Heparin is a highly sulfated glycosaminoglycan that binds to various proteins both in vivo and in vitro. Heparin interacts with exosomes [109]. Balaj et al. [110] successfully extracted cellular exosomes from cell culture media and human plasma using ultrafiltration and heparin affinity beads. In a recent study, heparin affinity chromatography has been shown to be a potential large-throughput method for separating different exosome subpopulations [111]. However, this method has poor specificity and is currently used only as an adjunct method.

Chromatography has long been of interest to researchers as an analytical method given its high throughput, high-purity output, high efficiency, and potential for automation. Separation can be achieved in a chromatographic column by packing a stationary phase with different affinities to the components to realize the difference in the distribution of the components between the 2 phases. Hydrophobic interaction chromatography is a suitable separation method for exosome extraction because of the different hydrophilicities of the components under mild conditions. Bruce et al. [112,113] recovered EVs from a variety of samples using polyethylene glycol terephthalate capillary channel polymer fibers and demonstrated the possibility of scaling them up to large-scale separations. Using this method, exosomes can be extracted in a shorter time and with fewer protein impurities. The packed columns can be reused several times, reducing cost and making the method more reproducible.

### Volume-difference-based separation techniques

In addition to density, volume is an important indicator that can distinguish exosomes from other impurities. Exosome separation techniques based on volume differences are also the first kind to be invented and matured. Among these, ultrafiltration and size-exclusion chromatography are the 2 most commonly used methods because of their unique and outstanding advantages.

#### Ultrafiltration

Ultrafiltration is a method of extracting exosomes based on molecular volume size; during the extraction process, the sample is sequentially passed through multiple ultrafine nanomembranes with cutoff values of 10 to 100 kDa, which ultimately separates it from impurities in clinical samples or cell culture media. This method is simple and efficient, not overly reliant on sophisticated equipment, and operational issues have less impact on the results. Most importantly, the ultrafiltration process is generally performed at room temperature and does not require the addition of additional reagents. Therefore, this method is less damaging to exosomes. Researchers have refined several ultrafiltration methods that can cope with different needs, including sequential filtration, tandem filtration, centrifugal ultrafiltration, and tangential flow filtration (TFF), which are briefly described in this section.

Both serial and sequential filtering are based on the same principle. Serial filtration involves the sequential placement of multiple screens with cutoffs between 20 and 200 nm in a syringe-like device [114], where cells and their debris are retained above the coarse screen; smaller substances such as proteins flow out through the 200-nm pores; and the desired exosomes are retained in an intermediate layer. In sequential filtration, individual filters within the device are individually housed in multiple devices, and the filtrate is added to the next device at the end of each filtration, with the desired exocytosis being left in the device after the last filtration is completed. The above 2 methods do not require cumbersome preparatory steps or complex instrumentation and, therefore, can be designed into various disposable filters or filter heads that are easy and quick to use. However, the pressure exerted during filtration may cause mechanical damage to the product, and the filter cake produced during filtration may clog the filter head, reducing the service life of the instrument and increasing the operating cost in the long run.

Centrifugal ultrafiltration, as the name suggests, is a technique that combines the application of centrifugation and ultrafiltration, utilizing centrifugal force to push the sample through the filter membrane and increase filtration efficiency. Prior to centrifugal ultrafiltration, the sample is usually subjected to rough filtration or centrifugation to remove large particles such as bacteria, cells and their fragments, or protein aggregates to ensure that ultrafiltration can proceed properly, which integrates the advantages of centrifugation and ultrafiltration, resulting in a purer product [115]. Unfortunately, this combination does not offset the disadvantages of both, and problems such as filter clogging and product inactivation persist. In addition, the extra steps increase the workload of the operator and professional competence is required.

TFF differs from the above filtration methods in that the fluid flows tangentially along the surface, avoiding cake formation, and thus reducing the incidence of membrane clogging. In TFF, the feed stream flows parallel to the membrane. By manipulating the hydrodynamic flow forces, the pressure exerted on the fluid causes only a portion of the fluid to pass through the membrane. As the membrane is constantly under parallel flow forces, potential clogging can be minimized by constant flushing [116] (Fig. S2C). The remaining retention can be recirculated back into the feed storage tank for repeated TFF [117]. As TFF has the potential for automation and high throughput, in addition to the advantages of reduced clogging, high reproducibility, and mild conditions [118], it has been widely used as an emerging technology for exosome preparation in clinical trials [119–121].

However, ultrafiltration separates components of different sizes, inevitably resulting in substances similar in size to the target exosome entering the product, thus reducing the purity of the elution. In addition, proteins adhering to the filter membrane and particles clogging the pores affect exosome elution. The pressurized operations of some ultrafiltration models can lead to mechanical damage to the target product, causing the extracted exosomes to lose their function.

#### Size-exclusion chromatography

In size-exclusion chromatography for exosome separation, a porous gel filtration polymer is used as the stationary phase, and the biological sample to be separated is used as the mobile phase. When a sample flows through a stationary phase with a porous structure, small-volume components enter the internal pores of the stationary phase and take a longer path out of the column. Larger components pass through the gaps between the stationary phases and exit the column more quickly than smaller components. The stationary phase or column can be filled with many gel polymers, including cross-linked dextran, agarose, polyacrylamide, or allyl dextran. Since the method is not pressure-driven, the resulting product is less mechanically damaged than the products of either ultra-isolation or ultrafiltration, providing a significant advantage in reducing vesicle rupture or activity reduction. Similar to TFF, size-exclusion chromatography has the potential for automation and large throughput. However, as with other chromatographic techniques, lower recovery volumes compel researchers to use more raw material, and because the method is unable to separate fractions that are similar in volume, additional extraction techniques such as immunocapture or polymerization precipitation may need to be used in the pursuit of high-purity exosomes.

### Polymerization precipitation

Similar to ethanol-mediated nucleic acid precipitation, hydrophilic polymers interact with the hydrophilic bonds of the sample exosome to form a hydrophobic microenvironment around the exosome, resulting in precipitation. Among various hydrophilic polymers, polyethylene glycol (PEG) stands out because of its low toxicity and cost. For separation, PEG, with a molecular weight of 6,000 to 20,000 Da, is generally used, and after pretreatment to remove large contaminants, the samples must be co-incubated with PEG solution; the hydrophilic polymer interacts with the hydrophilic bonds of the sample exosome to form a hydrophobic microenvironment around the exosome, resulting in precipitation. Because of its simplicity and ease of batch production, it has been favored by major biotechnology companies, which have launched a number of kits based on it, e.g., Total Exosome Isolation Reagent (Invitrogen, USA), ExoQuick (System Biosciences, USA), and ExoPrep (HansaBioMed, Estonia). A study by Niu et al. [122] on the isolation of exosomes from mouse uterine luminal fluid also demonstrated that this method achieves rapid isolation of exosomes while largely maintaining their physiological activity.

However, hydrophilic polymers can precipitate not only exosomes but also other structurally similar substances, such as nucleic acids, lipoproteins, proteins, and even viruses, and polymer precipitation is an early method used to extract viruses [123]. This method leads to pitfalls for the purity of the product and subsequent analysis, whereas in a side-by-side comparison experiment conducted by Gao et al. [124], the exosomes produced by this isolation method did not differ from those produced by the most basic ultra-isolation method in terms of bioactivity and integrity and were even slightly inferior in yield. Therefore, this method is mostly used as an adjunct to other methods.

However, recent studies have led to a turnaround in the approach. Antopolsky et al. [125] proposed a new method of assisted isolation of exosomes using Fe_3_O_4_ magnetic nanoparticles for preliminary exosome extraction. In this study, the addition of a layer of PEG-coated magnetic nanoparticles effectively reduced the concentration of impurity proteins in the feedstock, resulting in a significant increase in the proportion of particles with diameters of approximately 30 to 200 nm, the size of most exosomes, in the resulting product. In contrast, Paganini et al. [126] redesigned an efficient and stable precipitant from the source. This precipitant was based on the programmability of amphiphilic ionic polymers and was thus designed as a dynamic material for ion-exchange bioseparation. This novel material demonstrated satisfactory properties in subsequent applications and not only achieved a mild combination of aqueous 2-phase separations but was also used in chromatographic separation methods and demonstrated extreme programmability. This method holds promise for the flexible combination of polymerization–precipitation with other technologies, and the emergence of new polymerization agents can fundamentally compensate for the shortcomings of traditional polymerization and precipitation. With the advancement of related research, more polymerization agents with excellent performance will be developed. Polymerization–precipitation has gradually become an efficient and safe separation technique.

### Microfluidic technology

**Fig. 3.**
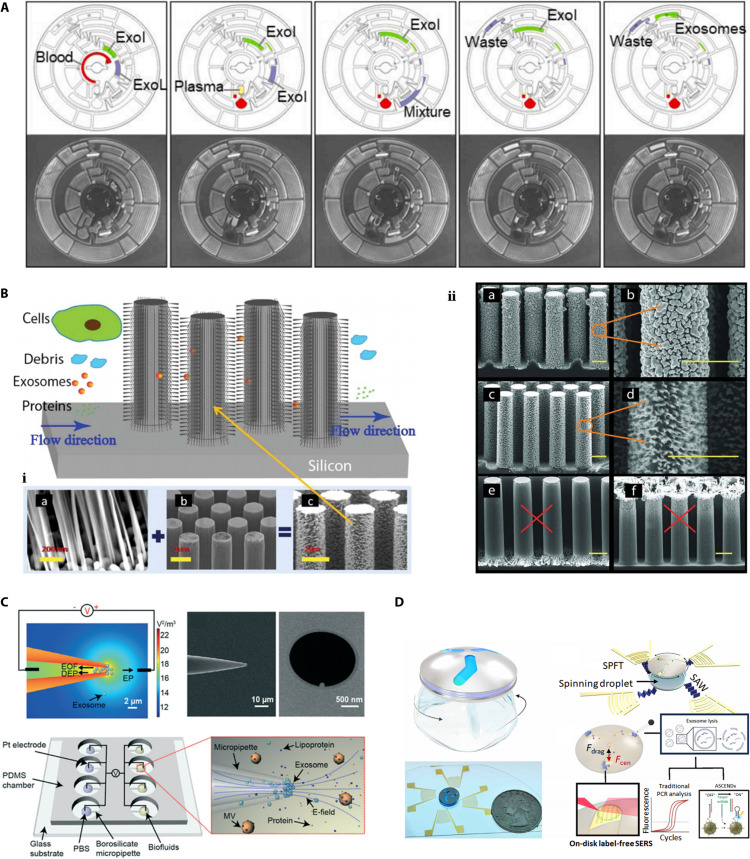
Various microfluidic chips for isolation of exosomes A) Schematic diagram of a microfluidic chip used to separate exosomes from plasma. Reproduced under terms of the CC-BY license. [128] Copyright 2022, Elsevier Ltd. B) Schematic diagram of the working principle of the ciliated microfluidic chip. Fluid with various components passes through the ciliated array, where larger materials are excluded, and target exosomes that bind specifically to the antibodies on the cilia are immobilized, while smaller and non-specific components flow through. i) and ii) represent the microstructures demonstrating two different cilia, respectively: i) (a) A representative porous silicon nanowire forest; (b) Micropillars; (c) Representative ciliated micropillars. The detailed fabrication process of the ciliated micropillars in ii) is shown in SEM images (a-f): (a, b) Uniform silver deposition on micropillar sidewalls via pulse-reverse plating, with (b) as a magnified view of (a); (c, d) Porous silicon nanowires formed by electroless etching, with (d) as a magnified view of (c); (e, f) Silver distribution varies with substrate resistivity, accumulating at the bottom for high-resistivity wafers (e) and near the tip for low-resistivity wafers (f). This hierarchical structure provides high surface area and tunable morphology for efficient affinity-based capture of exosomes. Reproduced with permission. [137] Copyright 2013, Royal Society of Chemistry. C) Schematic diagram of a microfluidic chip that relies on the principle of dielectrophoresis for the separation of exosomes. Reproduced with permission. [143] Copyright 2019, Royal Society of Chemistry. D) Schematic of a microfluidic chip that separates exosomes based on acoustic forces. Reproduced under terms of the CC-BY license. [146] Copyright 2024, American Association for the Advancement of Science.

Advances in micromachining and nanotechnology have led to the development of microfluidic devices, which have been combined with traditional exosome separation methods to derive a unique set of separation methods known as microfluidic separation technology, often involving the use of microchannels with dimensions of tens to hundreds of micrometers to manipulate small fluid volumes [127,128] (Fig. 3A). In 2010, Chen et al. [129] successfully extracted exosomes from plasma using microfluidics, which was the first attempt to apply microfluidic chips to the isolation of exosomes. Separation technology has taken a quantum leap with the introduction of microfluidics allowing researchers to utilize highly integrated chips for achieving accurate and efficient separation with small sample sizes and low analytical costs. Moreover, microfluidics can be easily integrated with the separation and detection of exosomes, which is crucial for clinical applications [130]. These separation methods are broadly categorized into volume-based, immunoaffinity-based, and noncontact separation microfluidics according to their separation principles.

#### Immunoaffinity-based microfluidics

Similar to other affinity capture technologies, designers must chemically modify the microfluidic chip to capture specific exosomes in a fluid containing a large number of impurities. Zhang et al. [131] developed a microfluidic exosome analysis chip using a novel graphene oxide/polydopamine. An ultrasensitive exosome enzyme-linked immunosorbent assay (ELISA) has been developed based on this chip.

However, nonspecific trapping still occurs in microfluidics. Vaidyanathan et al. [132] reported a simple method for improving the sensitivity of sensors by removing nonspecific adsorbed substances from the sensor surface using adjustable alternating current electrohydrodynamic forces. In the same year, the same team applied this technique to microfluidics and obtained expected results in capturing exosomes produced by cancer cells [133,134]. Previously, we discussed the problems of immunocapture, the most important one being the tight binding affecting subsequent elution. Therefore, after solving the nonspecificity problem of capture, separating the captured exosomes completely is another challenge for the researchers. Here, the affinity controllability of aptamer technology can play a role. Zhou et al. [135] applied designed aptamers to microfluidic technology and successfully and rapidly separated exosomes by recognizing CD63 and PTK 7 on their surfaces. This aspect not only affirms the advantages of aptamer technology in exosome capture but also reveals the possibility of microfluidics as a novel platform that can be flexibly combined with various other technologies. A specific anti-CD63 immobilized ciliated microcolumn separation technique designed by Qi et al. [136] could also solve this problem. In this study, large proteins flowed smoothly through ciliated column arrays, whereas exosomes were captured by cilia-specific binding. Finally, the captured exosomes were recovered intact by dissolving the cilia in a microcolumn and soaking them in phosphate buffered saline.

Overall, immunoaffinity-based microfluidics is fast, efficient, specific, and supportive of microdosing; however, continued efforts are needed to avoid nonspecific capture and maintain intact product elution.

#### Volume-based microfluidics

The commonly used volume-based microfluidic separation techniques mainly consist of the techniques of ultrafiltration and volumetric group-exclusion chromatography. The former is represented by ExoTIC, a technique that distributes a variety of membranes with pore sizes of 30 to 200 nm on a microfluidic chip, where membranes with a pore size of 200 nm are used to remove large vesicles or cellular debris, and membranes with a pore size of 30 nm are used to remove small-volume impurities from the product containing exosomes. This method is very efficient, and in the experiments of Liu et al. [101], this method has been shown to isolate exosomes with yields 4 to 1,000 times higher than the conventional ultracentrifugation method. In contrast, the nanowire exosome deblocking system, which is derived from volumetric group-exclusion chromatography, uses porous nanowires immobilized on a microcolumn as the stationary phase. As the sample passes through, the cells are blocked, and submicrometer particles, such as cellular debris, flow through the microcolumn layer unaffected. While particles with a particle size of approximately 30 to 200 nm are captured by the nanowire, and particles with different sizes have different retention times, different components can be separated in this way. Initially, nanowires were usually made of porous silicon [137] (Fig. 3B), but with the development of materials science, different types of nanowires have been invented. Chang et al. [138] successfully synthesized an Au@CuCl2 nanowire in a recent study. Ma et al. [139] developed a Cu(2)O-CuO@Ag nanowire by thermal oxidative growth and Ag nanoparticle sputtering and successfully completed clinical validation with serum specimens from patients with prostate cancer. In addition to updating materials inspired by the immunocapture method, Suwatthanarak et al. [140] designed peptide-functionalized nanowires. In this study, peptides screened for binding to the exosome marker CD9, based on the amino acid sequence of the EWI-2 protein, were immobilized on an array of ZnO nanowires. The results showed a significant increase in the exosome capture ability of the ZnO nanowires under the influence of peptide functionalization. These data provide ideas for nanowire technology to separate exosomes in combination with other techniques. Microfluidic separation techniques based on exosome volumes are highly efficient and support the manipulation of microfluids. There is no shortage of mature methods, such as ExoTIC, in this suite, and their combination with other technologies can compensate for the problems of the underlying methods.

#### Noncontact fluid technology

In all the above methods, exosomes must be in close contact with the nanochip, which could affect exosome integrity and the lifespan of the chip. In the next section, we introduce several noncontact microfluidic exosome separation techniques based on electrophoresis and acoustic force, which are different from traditional exosome separation techniques and may lead to new breakthroughs in exosome separation in terms of product integrity, reproducibility, and equipment lifetime.

##### Electrophoretic-based microfluidics

The phenomenon of dielectrophoresis was brought to the attention of researchers in the 1920s. It was first introduced by Pohl in 1951 [141] and has been sufficiently developed over the past decades to become a practical technique for the separation of cells, proteins, and various microscopic particles. In dielectrophoretic separation, a given sample in a nonuniform electric field is polarized and subsequently shifted by the dielectric force. At this point, larger particles, such as cells, are shifted to the low-electric field region, whereas nanoscale particles, such as exosomes, are attracted to the high-electric field region, resulting in the separation of exosomes. In the electric field, the size and electrical properties of the particles, electrical properties of the dielectric, and frequency and intensity of the applied electric field are the main factors affecting the dielectric strength. Thus, exosomes can be specifically captured from complex samples by controlling the frequency or intensity of the electric field. Barik et al. [142] successfully captured vesicles in a solution by a combination of site-selective dielectrophoresis and Raman spectroscopy and spectroscopically analyzed them simultaneously. This technique has been used to extract exosomes from biological samples. Shi et al. reported a novel dielectrophoresis device based on a series of borosilicate micropipettes with insulators and successfully achieved rapid separation of exosomes from cell culture media, plasma, serum, and saliva. This process requires only 20 min and is far more efficient than the ultra-isolation method, the gold standard for exosome separation [143] (Fig. 3C). In addition, the technique can be combined with ELISA, thus enabling rapid collection of assays from blood samples, which is more in line with clinical needs. Park et al. [144] proposed an integrated separation-analysis cancer diagnostic platform based on the dielectrophoresis-ELISA technique, which is 3 orders of magnitude more sensitive than the conventional ELISA method. Subsequently, the team validated the method using whole blood from model sources of breast, colon, and lung cancers, and the results showed that the accuracy of the method exceeded 95%.

The main problem with this technique is the generation of Joule heat when an electric field is applied. Currently, the main solution is to avoid this problem by designing better electrodes, and a few other methods have been reported in recent years [145]. Replacing the medium with a high-performance medium or controlling the ambient temperature may be directions worth investigating. However, at this stage, many studies have demonstrated that dielectrophoresis is a promising method because of its high efficiency and surprising accuracy, in addition to its ease of translation into clinical testing.

##### Acoustic microfluidics

The basic principles of acoustic microfluidics are as follows: Larger particles in a sample are subjected to a greater force of acoustic radiation, thus migrating faster toward the pressure node; the reverse is true for smaller particles. Generally, the separation occurs in roughly 2 steps: first, large particles such as cells and platelets are removed from the sample, leaving behind small particles such as exosomes and apoptotic vesicles. In the next stage, these particles are finely separated [146] (Fig. 3D). In a study by Naquin [146], exosomes obtained from this fine separation could be analyzed with 95.8% sensitivity and 100% specificity for the diagnosis of CRC. This technique was first used in the field of cell isolation and has matured, but only recently has it been put into active research in the field of exosome isolation; therefore, many problems remain to be solved. In fact, real solution environments are extremely complex, and their particles are often affected by a range of factors such as acoustic pressure, flow rate, and tilt angle. In addition, acoustic-based separation is a size-based separation technique that is still affected by impurities of the same size. To overcome the effects of these factors, Peng et al. [147] suggested that particle deflection could be improved by decreasing the input flow rate or increasing the acoustic pressure and acoustic frequency. Han et al. [148] reported an acoustic–fluidic separation chip, including a piezoelectric device that generates surface acoustic waves at a tilted angle and permanently bonded poly(dimethylsiloxane) microchannels and quantitatively analyzed the separated samples by a digital light scattering technique and flow cytometry validation; the results showed that the maximum purity of the separated particles could reach 95%.

As shown in the above study, this technique has a very high separation output purity, and the contact-free mode of separation ensures that the isolated exosomes retain their original structural integrity and biological activity. Since this technique was first used at the cellular level, it can be utilized directly in clinical samples, such as blood, under slightly controlled conditions. Although problems persist, this technology may have a substantial impact on clinical diagnostics.

Microfluidics is fast and efficient and is required for applications such as clinical diagnostics and laboratory extractions. The microchip design makes it easy to combine with a single technology, but it can also be integrated with multiple technologies to form a powerful, efficient, and versatile exosome analysis system that is not limited to separation. Many studies have demonstrated the possibility of combining it with a variety of other technologies; therefore, we can consider it a technology with great potential [149]. However, despite the undeniable advantages of microfluidic technology—such as rapid separation speeds and high product quality—the overly complex manufacturing processes and high costs remain marked technical hurdles preventing the large-scale production and clinical adoption of various microfluidic chips. Yet, high-precision 3-dimensional (3D) printing technology offers a promising solution to this challenge. High-precision 3D printing technology can directly convert digital models into complex 3-dimensional microstructures, significantly shortening the prototype development cycle. It is particularly suitable for the rapid fabrication of customized, small-batch chips. Simultaneously, for certain diagnostic applications, exploring the use of low-cost materials like thermoplastics and employing simple methods such as laser cutting and thermal imprinting for batch manufacturing of disposable chips represents an effective approach to lowering the threshold for clinical adoption. In the design phase, modular and standardized approaches—such as establishing libraries of microfluidic functional modules (e.g., standardized mixers, separators, reaction chambers, and detection zones)—allow researchers to assemble designs like building blocks according to specific needs, minimizing redundant development.

In summary, microfluidic technology is advancing toward higher integration, greater intelligence, and broader accessibility by continuously addressing core challenges like specificity, purity, stability, and accessibility. Although precision fabrication and operational complexity remain constraints, its unparalleled efficiency and integration advantages will drive ongoing innovation, ultimately yielding mature, reliable, and widely accessible next-generation exosome separation and detection platforms.

### Summary

Exosome isolation techniques, serving as the foundation for their safe and effective deployment across diverse applications, have gradually refined several classic and reliable methods over extended development. For instance, ultrafiltration—the earliest technique adopted—remains in use today due to its robust sample processing capacity and minimal equipment requirements. However, overly simplistic operational procedures and ambiguous identification criteria for exosomes result in suboptimal purity with this method. Methods like ultrafiltration and size-exclusion chromatography, which utilize exosome size characteristics as screening criteria, have also gained widespread adoption due to their efficiency and simplicity. Nevertheless, these approaches often struggle to precisely separate impurities of similar size, posing potential limitations in practical applications. A series of techniques derived from the immunological affinity principle have addressed this issue by enabling precise capture. Conversely, overly complex workflows and post-binding dissociation techniques hinder widespread adoption of these methods. Despite limitations, these approaches remain the primary methods for large-scale, straightforward exosome acquisition. Among them, ultrafiltration stands out for its stability and efficiency, earning the esteemed title of “gold standard”.

However, recent advancements in novel technologies have driven rapid and revolutionary progress in exosome separation techniques. Aptamer technology, derived from traditional antibodies, offers a novel solution for immunological affinity-based separation methods through its exceptional editability and controllable binding capacity. However, its complex structure undoubtedly poses challenges in design and synthesis. Concurrently, microfluidic technology—noted for its high throughput, sensitivity, integration, and rapid response—has garnered significant attention since its emergence. Nevertheless, the inherent complexity of these technologies imposes significant demands on production design and maintenance. Consequently, the future development of exosome isolation techniques can be broadly categorized into 2 parallel paths requiring simultaneous advancement: On one hand, these emerging technologies undoubtedly form the foundation for achieving more efficient and higher-purity exosome separation in the future. Therefore, continued in-depth research is essential, alongside addressing the shortcomings of existing techniques. On the other hand, researchers must also focus efforts on scaling up production and ensuring quality control. This will accelerate the adoption of these promising new technologies in both research and clinical settings, transforming theoretical advancements into practical applications.

## Enhanced Exosome Construction

The above cited studies have proved the various advantages of exosomes as an emerging drug system, and simultaneously, they have revealed various shortcomings of the application of exosomes, such as insufficient targeting, easy decomposing, and inability to meet the efficacy standards, as theoretically expected, owing to the low content of active ingredients. Obviously, the various potentials of exosomes have not been fully stimulated at this stage. In this chapter, we focus on the efforts made by researchers to compensate for the deficiencies of exosomes, focusing on drug delivery and drug potency enhancement, and hope to explore a series of practical exosome enhancement technologies that will open the way for the application of exosomes in a broader platform.

### Enhanced drug delivery

#### Enhancement of targeting

The specificity of exosomes is mainly derived from various membrane proteins on their surface, and most existing enhancement-targeting techniques are based on this approach. Exosomal membrane proteins can be altered by strategies such as self-expression in the mother cell, modification of existing proteins on the membrane surface, and splicing of foreign membranes. Three major modification methods have been developed: genetic engineering, chemical modification, and membrane fusion.

##### Genetic engineering

Genetic engineering technology, after a long period of development and iteration, has become a mature and easy-to-execute technology and is the most commonly used means of modifying exosome targeting. In genetically engineered exosomes, the target gene is first combined with a carrier, such as a plasmid, to form a recombinant plasmid. Subsequently, the recombinant plasmid is introduced into the mother cell. When the mother cell successfully expresses the target gene products and secretes exosomes carrying these products, it marks the success of genetic engineering modification. The target gene is usually templated with a nucleic acid sequence that expresses a protein that the exosome itself expresses; therefore, it is modified to express a more targeted protein.

LAMP-2B is a common surface protein. This protein belongs to the lysosome-associated protein family and is abundantly expressed on the surface of exosomes produced by DCs. A C-terminus firmly anchored to the inner side of the membrane and an exposed and bulky N-terminus are the main reasons for the widespread interest in this protein. These structural features allow the moiety modified at the N-terminus to be naturally exposed for targeting functions, making it a good target for modification. Functional modification of LAMP-2B with an iRGD cyclic peptide with high affinity for cancer cells is another proven approach. Liu et al. [150] engineered immature DCs to secrete exosomes with iRGD peptide functional modification of LAMP-2B protein on the surface to achieve targeting of OCI-Ly8 cells that highly express the integrin αvβ3 receptor. Wang et al. [151] expressed an exosome of the LAMP-2B protein with an iRGD peptide functionalization modification on the membrane surface using engineered human embryonic kidney (HEK)-293T cells. Follow-up experiments demonstrated that this exosome had a high affinity for cancer cell surface integrins and inhibited tumor development significantly without side effects in the 8505C xenograft mouse model. In addition, Liang et al. [152] constructed a target-enhancing exosome for targeted delivery to chondrocytes by genetically fusing a chondrocyte-affinity peptide to the N-terminal end of Lamp2B. Xu et al. [153] combined the MSC-binding peptide E7 with LAMP-2B to obtain an exosome displaying the E7 peptide on the surface and used the exosomes to successfully target the delivery of kartogenin, a small molecule drug that induces SF-MSC differentiation into chondrocytes in vitro and in vivo, to the affected area. Alvarez-Erviti et al. [154] genetically engineered a target-enhancing exosome that expressed LAMP-2B and carried small interfering RNA into the mouse brain.

CD63, the most common 4-spanning protein superfamily on the surface of exosomes, can also be used as a tool to produce targeting–enhancing exosomes. Liang et al. [155] obtained engineered exosomes carrying functional miR-26a and targeting inhibition of HepG2 cell proliferation and growth by loading drugs through the electroporation method via a gene fusion between the transmembrane proteins of CD63 and the sequence of Apo-A1. Gao [156] used CP05 to modify CD63 on the surface of exosomes and validated its targeting ability in a mouse model.

Genetic engineering is a trusted technology whose reliability has been proven over the past few decades. Exosome modification can be referenced from existing technology and experience, and the selection of genes and subsequent modification treatments become traceable, greatly reducing the workload of researchers. However, the treatment conditions of this technology are mild and cannot easily affect the activity of exosomes. Most importantly, the modified donor cells can continue to produce the required exosomes in a continuous stream, which, in turn, reduces the cost of subsequent production. However, modification at the genetic level remains a considerable challenge, and it is questionable whether the base sequences loaded into the cells can be successfully expressed. Cell traits produced through genetic engineering are often not stably retained, and establishing stable cell lines still requires a lot of work. However, this remains the most commonly used method for exosome modification. In the long run, the sustainability of cells produced using this technique is expected to have more advantages in terms of mass production.

##### Chemical modifications

The modification of existing proteins on the surface of exosomes is likely to be the most direct approach. Some small molecules that can be recognized by specific cells have good stability and small space occupation are the most commonly used raw materials for modification. Zhang et al. synthesized a neutrophil exosome functionalized with ultrasmall Prussian blue particles with excellent targeting and anti-inflammatory properties. These exosomes selectively recognized activated fibroblast-like synoviocytes and progressively accumulated them for sustained action [157]. Sialic acid is widely distributed on the surface of nerve cells and is a major constituent of the mammalian brain [158]. Using exosomes functionalized with sialic acid and its analogues, drugs can be targeted to neuronal cells. Zheng et al. [159] synthesized 5 sialic acid analogues with different lengths of N-acyl side chains that can be used for exosome modification and used them to process macrophages to produce an exosome with a high affinity for microglia.

In addition to a variety of small molecules, peptides with targeted functions can be implanted into or bound to the exosome membranes. The cRGD peptide (cRGD) is a tripeptide sequence containing arginine–glycine–aspartic acid (Arg-Gly-Asp), which is specifically recognized by integrins [160]. Integrins are rarely expressed in normal cells but are widely distributed on the surface of cancer cells, providing a theoretical basis for cancer cell targeting of cyclic RGD peptide-functionalized exosomes. A number of cRGD peptides targeting αvβ3 integrins have been identified, such as cRGDfC, cRGDfK, cRGDfE, cRGDfV, and cRGDyV [161]. The discovery of these peptides indicates the application of cyclic RGD peptides. Among them, cRGDyK is the most widely used one. Tian et al. [162] pioneered the use of a simple, rapid, and biologically orthogonal chemical reaction to couple cRGDyK peptides with exosome surfaces (Fig. S3A). In a mouse model of transient middle cerebral artery occlusion, the exosome successfully delivered loaded drugs to the ischemic lesion area of the brain. KDEL peptides from the endoplasmic reticulum can also be used for exosome modification. Wang et al. [163] used exosomes modified with KDEL peptides from cow’s milk to deliver both PD-ligand 1 (PD-L1) siRNA and celastrol to promote chemoimmunotherapy in a recent study. KME demonstrated superior targeting to controls.

Peptide or protein functionalization modifications exhibit excellent targeting properties; however, their structures, especially the spatial structures of proteins, are susceptible to various factors and difficult to maintain. In contrast, the aptamer technology mentioned previously is a more suitable scheme for enhancing exosome-targeting function, and loading aptamers into exosomes confers increased cellular selectivity. More importantly, the aptamer, as a shorter nucleic acid fragment, can be self-edited and designed with less difficulty, thus possessing more possibilities for practical manipulation. Song et al. [108] identified 2 DNA aptamers, CD63-1 and CD63-2, with high affinity and specificity for CD63 proteins, of which CD63-1 can be used against CD63-positive cells including breast cancer MDA-MB-231 cells and CD63-overexpressing HEK293T cells.

Despite their simplicity, directness, and numerous options for chemical modification, they have not received marked attention. The main reason for this is that a relatively harsh chemical environment often causes structural damage or even inactivation of exosomes. The purification of exosomes after the reaction is also a difficult operation, which can lead to a decrease in the yield and purity of the exosomes or even the addition of toxic impurities. Therefore, the use of less toxic and more easily separable raw materials and the search for milder reaction conditions are the main directions for improvement of the method. In addition, new purification and separation techniques that are highly efficient but mild may be the most urgent need for the chemical modification method. With the development and improvement of this field, the chemical modification method may be able to realize its full value earlier.

### Strategies for functionalizing exosomes

#### Membrane engineering

The nature of different exosomes varies greatly, and their contents vary even more. However, they all share a common basic structure, the most important of which is a fluid lipid bilayer-based membrane. This aspect provides a theoretical basis for the generation of new functionalized exosomes through membrane fusion; some exosomes or designed liposomes with excellent targeting but no obvious therapeutic effect will be useful. Below is a brief introduction to commonly used membrane fusion technologies and their latest developments, with the principles and advantages and disadvantages of each technology summarized in Table S1.

##### Membrane fusion materials

Liposomes are the most common membrane fusion materials. Liposomes are simple in structure, have the same lipid bilayer as exosomes, and can be easily modified. These advantages have made them popular tools for targeted drug delivery, and in recent years, many experiments have demonstrated that liposomes can be membrane-fused by various means [164,165]. In addition to liposomes, the fusion of some functional cell membranes is another strategy for enhancing exosome targeting. For example, erythrocyte membranes have the ability to target hypoxic brain tissues, and Liu et al. [166] utilized this property to obtain a nanovesicle capable of targeting and protecting hypoxic brain cells by fusing erythrocyte membranes and platelet membranes encapsulating the hypoxia-inducible factor-1α inhibitor YC-1 (Fig. S3C). Xie et al. [167] targeted platelet membranes to atherosclerotic plaques and designed a transplatelet membrane-modified M2 macrophage exosome. High targeting alleviates atherosclerosis in LDL receptor-deficient mice via intravenous injection, and macrophages, as a component of the body’s immune system, can recognize and phagocytose pathogens. Their cell membranes and exosome membranes inherit this property and can also be used as synthetic materials for enhanced targeting of exosomes. Rayamajhi et al. [168] successfully obtained a hybrid vesicle with homogeneous particle size for cancer cell targeting using exosomes from mouse macrophages as the raw material.

Some cancer cells and the exosomes they produce can achieve mutual recognition, which may be related to their homing effects [169]. Inspired by this principle, Zhang et al. [170] synthesized a novel artificial chimeric exosome by selecting membranes derived from erythrocytes and MCF-7 cancer cells and utilizing their respective characteristics. This exosome possesses both the anti-phagocytosis ability of erythrocytes and the targeting ability of cancer cells, which ensures that the exosome will not be cleared by the body and can simultaneously transport the drug to the affected area effectively. Zhou et al. [171] removed all exosomal contents isolated from hepatocellular carcinoma cells, retained only their exosomal membranes, and subsequently constructed a novel hybridized vesicle with phospholipids for targeted delivery of therapeutically effective siRNA. This vesicle could bypass the endosomal degradation pathway and increase the siRNA transfection efficiency by 1.7-fold while maintaining high targeting. However, the cell is a heterogeneous system, and even the contents of exosomes, which are secretions, are so diverse that it is very difficult to obtain pure cell membranes. In the direction of membrane binding, most researchers still choose to directly bind these vesicles to each other. However, many risks are associated with this behavior, especially when hybrid exosomes are based on the membranes of cancer cells and their derived exosomes, where the surface composition is more complex and the biological mechanisms are less well defined, making them susceptible to safety issues. However, the superiority of cancer cell membranes cannot be ignored. To resolve this contradiction, Vázquez-Ríos et al. [172] attempted to introduce liposome technology and designed an exosome-mimicking nanosystem. In this study, the membrane composition was tightly controlled while preserving the structure and function of the exosome membranes of tumor cells. There is still much room for the development of membrane-binding technologies based on cellular or exosomal membranes, and perhaps, this mimetic membrane system is a worthwhile research direction in this field.

##### Co-incubation

**Fig. 4.**
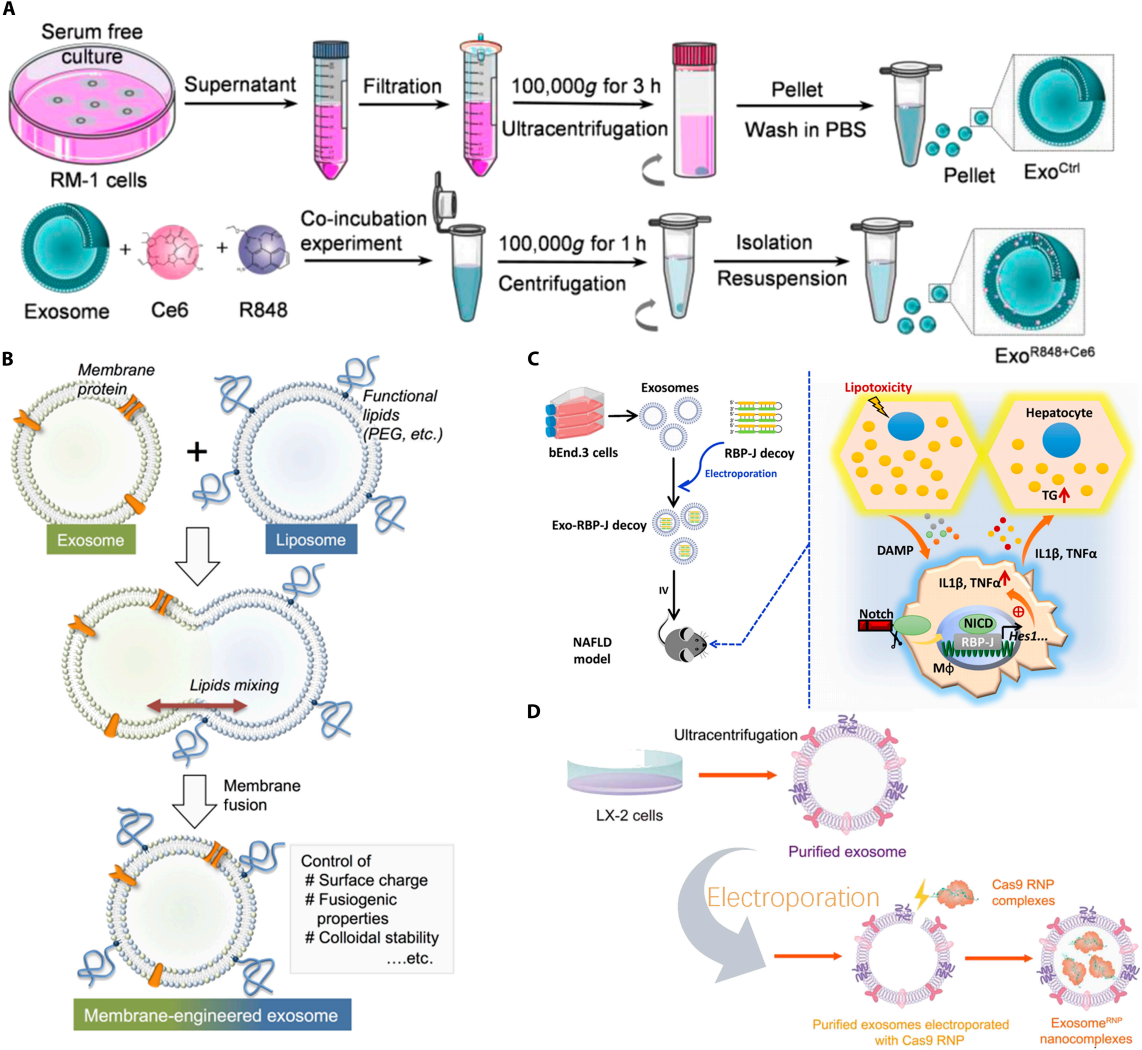
Exosome modification. (A) Schematic diagram of exosome modification by means of co-incubation: target exosomes were isolated from cell culture fluid using ultracentrifugation and subsequently mixed with surface modification molecules for co-incubation. Reproduced under terms of the CC-BY license [173]. Copyright 2022, Taylor & Francis. (B) Schematic diagram of exosome modification by means of membrane fusion: exosomes and liposomes are mixed thoroughly, and then the freeze–thawing method is used to destabilize the original membrane of the two to ensure fusion of the 2 membranes. Reproduced under terms of the CC-BY license [181]. Copyright 2016, Nature Portfolio. (C) Schematic diagram of exosome modification by means of electroporation: instantaneous high current is applied to the exosome, and the drug enters the exosome under the conditions of membrane instability. Reproduced under terms of the CC-BY license [207]. Copyright 2023, Ivyspring International Publisher. (D) Schematic diagram of exosome modification using electroporation combined with genetic engineering: instantaneous high current is used to destroy the stability of the exosome membrane, and the gene editing tool Cas9 is smoothly loaded into the purified exosome. Reproduced under terms of the CC-BY license [208]. Copyright 2022, American Association for the Advancement of Science.

The lipid bilayer membrane of exosomes spontaneously fuses with other membrane structures. Therefore, mixing and co-incubating exosomes from 2 different sources may be the simplest way to obtain hybrid exosomes [173] (Fig. 4A). Lin et al. [164] obtained hybrid exosomes by co-incubation (Fig. S3B). This exosome can deliver the CRISPR-Cas9 system in MSCs when being endocytosed by MSCs, expressing encapsulated genes. Zhang et al. [174] developed an exosome that enables targeted pathological angiogenesis therapy by co-incubation with exosomes produced by macrophages. Guo et al. [175] focused on the stability of the internal environment of exosomes; they successfully loaded the photosensitizer indocyanine green, which is poorly stable and has a short plasma half-life, onto ginger-derived exosome-like nanoparticles. The engineered exosome-like particles obtained by this approach could be readily taken up by cells via a lipid-dependent pathway, and satisfactory results were obtained in subsequent light-mediated therapeutic tests. However, Zhao et al. noted that the drug concentration of methotrexate is crucial in the treatment of central nervous system (CNS) lymphomas. A high drug concentration is required for effective tumor inhibition. Therefore, the team successfully designed a highly targeted engineered exosome and encapsulated methotrexate in the exosomes via co-incubation. Subsequent high-performance liquid chromatography assays showed that the method was successful and efficient for loading large amounts of MTX, as expected [176].

This co-incubation method is simple, convenient, and does not require delicate manipulation or complex tools. However, spontaneous membrane fusion is a lengthy process, probably because most liposomes have a negative ζ-potential to ensure their stability in the bloodstream, while exosomes are usually negatively charged. This makes the co-incubation method extremely unfavorable in terms of electrostatic interactions. Some lipid-soluble drugs escape from exosomes during this process, which further affects product availability.

##### Co-extrusion

The solution to several problems caused by the long time required for co-incubation fusion is to overcome the repulsive forces between particles. One option is to apply an external mechanical force to accelerate membrane fusion. When particles of different origins with a lipid membrane structure pass through a small pore under an external force, an otherwise stable membrane structure is affected. In this case, these particles tend to form multiple uniformly sized particles, a treatment known as co-extrusion. Utilizing this method, Ducrot et al. [177], in their recent study, mixed platelet membranes and MSC exosomes in equal amounts and produced vesicles with homogeneous particle sizes by passing them through 400- and 200-nm polycarbonate porous membranes multiple times under external force. Subsequent experiments demonstrated that these vesicles perfectly inherited the ability of targeting platelet membranes to the damaged myocardium as well as the pro-angiogenic ability of MSCs, and the results of membrane fusion fully met the expectations of the researchers. Ji et al. [178] developed an exosome–liposome hybrid drug delivery system co-loaded with clodronate and nidaneb. The team co-extruded a mixture of liposomes and exosomes at a ratio of 5:1, and fusion vesicles with a diameter of 110 nm were isolated and purified, demonstrating excellent targeting, anti-inflammatory function, and anti-fibrotic effects in a mouse model of pulmonary fibrosis.

Co-extrusion is a simple and fast processing method that consumes significantly less time than co-incubation. It is also less variable and reproducible than other methods, with the potential for large-scale production. However, the mechanical force exerted on the raw material during operation not only promotes membrane fusion but also inactivates, deforms, and even ruptures some of the particles, and the integrity of the product is not guaranteed. In addition, the contents of ruptured exosomes are dispersed in the system in large quantities, which may lead to product adhesion. All these problems have a considerable impact on the yield and subsequent purification. Therefore, this method is not recommended when preparing a small number of samples and when the conditions for other fusion methods are met.

##### Membrane destruction–reconstruction-based membrane fusion techniques

When utilizing ultrasound to induce exosome separation, prolonged processing often results in the disruption of the original membrane structure. The disrupted lipid fragments are highly unstable and tend to bind to each other after a few moments of standing. Ultrasound-induced membrane fusion utilizes this property to produce hybrid exosomes.

Cheng et al. [179] repeatedly sonicated genetically engineered exosomes of CD47 derived from tumor cells and exosomes from M1 macrophages to obtain a novel hybrid exosome that could activate the cyclic guanosine monophosphate–adenosine monophosphate synthase/interferon gene. Because of the presence of the D47 protein on the surface, this exosome possesses a high degree of tumor cell selectivity along with an extremely long somatic circulation time. Jang et al. [180] mixed ultrasound-treated pancreatic cancer cell exosomes and lipid-coated microbubbles and successfully obtained a highly targeted hybrid exosome that could be used for both PDT and immunotherapy using reverse evaporation.

A principle similar to ultrasound induction is the freeze–thaw method. Freeze–thaw is a common means for obtaining cell membranes; at low temperatures, ice crystals are formed that cut through the cell, and the temperature is then raised, and the ice crystals melt while the whole membrane turns into highly unstable fragments. Upon rewarming, the broken membranes begin to combine to form new stable membranes [181] (Fig. 4B). Researchers soon realized that this approach is also applicable to the fusion of exosomes or liposomes and put it into practice. Sato et al. [181] collected exosomes produced by Raw 264.7 cells and successfully developed a hybrid exosome by freezing it with liposomes under liquid nitrogen and incubating it at room temperature for 15 min. After verifying the feasibility of the freeze–thaw method in the direction of exosome membrane fusion, the same experiments performed using exosomes produced by CMS 7 cells overexpressing the human HER 2 receptor demonstrated that exosomes produced by genetically engineered modified cells can also be used to produce hybrid exosomes using the freeze–thaw method.

However, in subsequent experiments, the freeze–thaw method proved to be unsuitable for the binding of exosomes to hydrophilic drug-carrying liposomes [177], and the feedstocks that could be used for this method were further restricted. However, a recent study by Cheng et al. [182] may bring a turnaround to this situation. They found that by combining drug-loaded thermosensitive liposomes by freeze–thawing, the hybrid exosomes obtained by fusion not only maintained an acceptable drug-loading capacity but also executed PTT under laser irradiation by loading with photothermal agents after intravenous administration.

The sonication-induced and freeze–thaw methods almost completely discard the integrity of the original membrane, and the membrane fragments produced by sonication and ice crystal formation vary in size, leading to large differences in the recombinant exosomes produced by this method; this may cause problems in subsequent applications, especially for precision instruments, such as microfluidics, which require other separation and purification techniques to optimize the products. However, for some experimenters who do not require high product size and or need to load exosomes, these 2 methods remain simple and reliable.

##### Potential-guided membrane fusion

As mentioned previously, the greatest difficulty in membrane fusion is the repulsion caused by the same potential. Most of the previously described methods involve external forces that weaken the original stability of the membranes, thereby facilitating their fusion. However, if 1 of the 2 membranes to be fused can be positively charged by an appropriate means, the original repulsive force will become the driving force for the combination of the two, and the 2 most pressing problems of poor spontaneity of membrane fusion and rupture of the membrane due to the application of an external force will be solved.

This idea was initially applied to cartilage therapy, where conventional exosome treatment strategies had low bioavailability owing to the similarly negatively charged cartilage matrix. Feng et al. [183] attempted to modify small EVs derived from MSCs with a novel cationic amphiphilic macromolecule, ε-polylysine-polyethylene-distearyl phosphatidylethanolamine. The modified cell vesicles had their charge flipped, and their affinity for the cartilage matrix was significantly increased. Inspired by this, Piffoux et al. [165] successfully bound positively charged liposomes to human umbilical vein endothelial cell-derived exosomes using ethylene glycol as a triggering agent. The binding method proved to be 20% to 30% more efficient than conventional methods. This method retains the integrity of the exosome, which is almost intact, and cleverly defuses kinetic problems encountered during membrane fusion. The by-products and impurities generated during the modification process can also be separated prior to the membrane fusion operation, thus not affecting the product purity, but making it a promising membrane fusion method.

Among the above schemes for membrane fusion, co-incubation is the simplest and easiest; however, the method requires a long reaction time and is susceptible to various factors, and the product quality is not stable. The fusion rates of the ultrasound-induced and freeze–thaw methods were much higher, and the conversion rates were also improved. However, ultrasound induction and freeze–thawing methods are based on the complete fragmentation of the original membrane; therefore, it is impractical to completely ensure the integrity of the original exosome membrane and contents. Simultaneously, the fragmented and recombined exosomes cannot achieve uniformity in size, requiring methods such as co-extrusion or purification assistance. In contrast, potential-guided membrane fusion techniques do not require external stimulation and can be performed spontaneously. However, both the electrode conversion of exosomes and the search for oppositely charged lipid membranes are difficult processes, and the development of this technique may depend on efforts in these directions. None of the above technologies are amenable to large-scale production, in contrast to co-extrusion, which is probably the one with the greatest potential for large-scale production. Unfortunately, microfluidics, which have the potential for high throughput and automation and are widely used in exosome processing, have not yet been widely used in membrane fusion. However, at present, production techniques that combine multiple technologies may represent the optimal solution to address the various challenges arising during the modification process of exosomes [184,185] (Fig. S3D and E).

This could be the focus of development in the field of exosome membrane fusion modification in the next decade or so, and we look forward to the emergence of a new generation of microfluidic chips with strong membrane fusion processing capabilities. In addition, the increased targeting of exosomes will not only bring about developments in their own applications but also inspire advances in separation technologies, especially those based on affinity capture. We expect exosome-specific capture technologies to substantially improve the exosome capture accuracy in the near future, thereby creating opportunities for obtaining large quantities of high-quality exosomes.

##### Enhancement of exosome uptake

Various exosomes enriched toward the affected area must be further absorbed to achieve cellular therapy, which is mainly accomplished by membrane fusion. Membranes from homologous cells are more readily accepted than heterologous ones. Sun et al. [186] designed a permeation-enhanced exosome delivery protocol using fibroblast-derived exosomes based on their specific affinity for homologous exosomal fibroblasts. Following transnasal administration, the EL-CLD hybrid system preferentially accumulated in fibrotic lungs, significantly increasing permeability within fibrotic lung tissues through targeted delivery and substantially increasing drug bioavailability. In addition, phagocytosis of exosomes by target cells is one of the pathways by which exosome contents enter the cell interior, which is more efficient than passive membrane fusion. The key to enhanced cellular uptake of nanoscale particles lies in specific molecules originating mainly from the cell surface. Cx43 is an important connexin protein that plays an important role in cellular transcription, metabolism, autophagy, ion channel transport, and intercellular communication [187]. Cx43 forms functional channels on the surface of exosomes, thereby promoting the cellular uptake of nanoscale vesicles [188]. Chai et al. [189] elucidated the importance of Cx43 in cancer cell therapy. In the tumor environment, leukemia-derived MSC in vivo convert adjacent MSC into a permissive state that promotes leukemia cell proliferation, survival, and chemoresistance. Using a reverse engineering approach, the team explored how the leukemia BCR-ABL1-driven exosome-miR130b-3p interacts with gap junction Cx43. Inspired by this, Valiunas et al. [190] designed a protocol based on the delivery of oligonucleotides of specific genes or genetic programs to cancer cells via Cx43 to induce apoptosis and thus cause effective tumor suppression. In their study, they successfully synthesized in vitro toxic oligonucleotides targeting 3 cancer cell lines and subsequently expressed Cx43 by stable transfection for therapeutic purposes. Cell-penetrating peptides are short-chain hydrophobic peptides that enhance the uptake of different cargo molecules across eukaryotic cell membranes by promoting endocytosis [191]. Jing et al. [192] loaded a cell-penetrating peptide onto milk exosomes and succeeded in obtaining an engineered exosome that effectively alleviated ulcerative colitis in mice.

The antimicrobial protein CAP18, an active protein comprising a negatively charged LPS-binding active C-terminal fragment, was first isolated from rabbit granulocytes. Noguchi et al. [193], in a recent study, found that the (sC18)2 peptide derived from CAP18 possesses the biological function of inducing cytosolic drinking. Furthermore, in loading experiments with exosomes, after a simple modification of its hydrophobic segment, the derived peptide was able to bind readily to exosome membranes while retaining its potent pro-macrophage phagocytic function. They then used modified exosomes for the cytoplasmic delivery of functional toxin proteins for biocytosis, with satisfactory results.

Cellular uptake is an important part of drug action, and exosomes exhibit excellent plasticity and potential. However, the enhancement of exosome uptake is largely dependent on the identification and isolation of novel signaling molecules. Moreover, the loading difficulty of different signaling molecules varies. Some molecules show affinity for membranes after simple modification, whereas others may need to be stabilized and loaded using genetic engineering. Fortunately, after more than a decade of research on exosome modification, we have accumulated many tools; therefore, for enhancing the functionality of exosomes, especially enhancement of drug efficacy, uptake at the cellular level must be considered.

#### Content engineering

##### Expansion of exosome sources

Exosomes produced by human-derived cells have attracted considerable attention because of their low rejection, safety, reliability, and efficacy. At this stage, most studies focus on human-derived exosomes, especially DCs, macrophages, mesenchymal stem cells, and other cells that play an important role in immunity and wound healing and can produce exosomes that can be easily detected in body fluids and plasma.

In contrast, Watanabe et al. [194] focused on osteoblastic exosomes, which are minimally distributed in plasma and easily overlooked. Prior to this, researchers had secreted and studied few skeletal exosomes owing to the lack of concise markers to distinguish skeletal muscle-derived exosomes from exosomes. In this work, they identified marker proteins that can effectively label skeletal muscle exosomes and, using this effective tool in subsequent experiments, successfully proved the conjecture that skeletal muscle-derived exosomes are present in very small quantities in plasma but are concentrated in the muscle microenvironment and are able to play important roles by releasing specific proteins and miRNAs. Leng et al. [195] also succeeded in extracting exosomes from muscle tissues that can attenuate muscle degeneration by stabilizing damaged muscle membranes. Therefore, the role played by exosomes in the microenvironment cannot be ignored.

Huang et al. [196] recently revealed another identity of skeletal muscle exosomes. Their study demonstrated that skeletal muscle-derived exosome miR-484 attenuated stroke-induced iron death in neuronal cells by reducing the production of lipid peroxides, up-regulating glutathione peroxidase 4 and solute carrier family 7 member 11, and down-regulating acyl coenzyme A synthetase long-chain family member 4. This implies that the cellular secretions that act in the microenvironment also influence behaviors such as the growth, development, and repair of the nervous system, suggesting the need for research and development of this class of exosomes for neurological and endocrine applications.

Cancerous tissues often exhibit abnormal cell proliferation and angiogenesis, which may be related to substances such as nucleic acids within the exosomes of cancer cells. Qiu et al. [197] pointed out that miR-519a-3p in the exosomes of gastric cancer cells might lead to the M2-like polarization of macrophages by targeting the activation of the MAPK/ERK pathway, thereby inducing angiogenesis. Subsequent experiments, such as tubule formation and aortic ring assays, confirmed the excellent effects of gastric cancer cell-derived exosomes in promoting angiogenesis.

Chen et al. [40] also observed a similar phenomenon in exosomes produced by colon cancer cells, but the pro-angiogenic pathways were not the same; colon cancer-derived exosomes essentially enhanced vascular endothelial cell migration and tube formation by inducing filamentous pseudopod formation and endothelial cell tilting. Notably, they also compared metastatic and nonmetastatic cancer cell exosomes in terms of pro-angiogenesis and identified miR-146b-3p as a key regulator of exosomes promoting angiogenesis in colon cancer cells. The exosomes produced by cancer cells seem to be a treasure trove of angiogenesis, and biologics derived from them could play a considerable role in wound healing, promoting tissue development, and so on. However, these experiments are still at the cellular level, and some biological components and their roles remain unclear. The right time to open this cellular level, and some trove of angiogenesis, and biologics made from them could play a considerable role in wound healing, promoting tissue development, and so on.

Exosomes from other animal and plant cells or body fluids can also be used for disease treatment. Because of their similar physiology, mammalian exosomes were the first group of exogenous biological vesicles to come to the forefront of attention. Castaño et al. [198] successfully extracted exosomes from the blood and muscle tissues of mice that could enhance insulin sensitivity by down-regulating hepatic FoxO1. Apart from various tissues and cells, animal body fluids are also major sources of exosomes. Among the many body fluids, milk was the first to be researched because of its advantages, such as high production and biocompatibility. In the last decade, its earliest role was similar to that of analogous liposomes in drug delivery [199]. Subsequently, its role in bacteriostasis [200], inhibition of oxidative stress [201], anti-cellular senescence [202], stimulation of osteoblast formation, and inhibition of bone resorption was explored [57]. Bovine or porcine serum from the neonatal or fetal period has also been shown to be useful for treating some viral infections [203].

Plant-secreted vesicles that are morphologically and functionally similar to exosomes are known as exosome-like particles of plant origin or plant exosome nanovesicles. These vesicles are involved in the alleviation or treatment of some diseases in humans as well. Zhou et al. [204] successfully extracted a lipid- and polysaccharide-rich plant exosome vesicle from the berries of *Lycium barbarum*, family Solanaceae; the cross-sectional area of quadriceps muscle and the expression of the AMPK/SIRT1/PGC1α pathway were significantly improved in a mouse muscle atrophy model after its intramuscular injection. In subsequent energy-targeted metabolomic analyses, this plant-derived exosome activated muscle regeneration through the up-regulation of quadriceps muscle amino sugar and nucleotide sugar metabolism, autophagy, and oxidative phosphorylation processes.

Plant-derived exosomes can also play an important role in the TME; Kim et al. [205] produced ginseng-derived plant exosome-like particles with high blood–brain barrier (BBB) and glioma-targeting properties, and these particles were effective in killing tumor cells by mass recruitment of M1 macrophages upon reaching the microenvironment of gliomas.

Sundaram et al. [70] recently found a special use for plant exosomes in addition to the direct action on the affected area. They successfully isolated exosomes from garlic and used the substance to train human intestinal *Akkermansia muciniphila* and obtain an outer membrane vesicle from a bacterium with a functional structure similar to that of exosomes. Subsequently, these exosomal vesicles entered the brain and were taken up by microglia via the oral route of administration, where they increased the expression of the insulin receptor substrates IRS1 and IRS2, thereby reversing high-fat diet-induced type 2 diabetes in mice. This novel and effective therapeutic approach of plant exosome training of gut bacteria and the subsequent regulation of gene expression in the brain via outer membrane vesicles provides new ideas for exosome conversion. This approach of training the intrinsic flora of the human gut is also safer and more acceptable to patients. With continuous improvements, this approach may become the most important application of exosomes for the treatment of this type of disease.

Expanding the source of exosomes can not only compensate for the functional deficiencies of existing exosomes but also allow us to combine newly discovered exosomes with existing exosomes to utilize the unique functional molecules on the membrane of the newly explored exosomes, resulting in exosomes with more functionalities. These exosomes can be used to explore the principle of action of internally loaded nucleic acids and proteins and to extract and load them individually into liposomes or other exosomes that have been proven to be safe and reliable. However, the use of exosomes, particularly exogenous and pathogen-derived, remains risky. As a xenobiotic substance, it can easily activate the immune system and be cleared quickly by the body, resulting in short-lived efficacy of the given drug. Furthermore, some exosomes can trigger hypersensitivity reactions, resulting in serious consequences. Therefore, in addition to functionality, the biocompatibility of exosomes is a matter of concern.

##### Exogenous loading

In the application of natural exosomes, the monotonous nature of their contents leads low-level therapeutic effects, and therefore, by artificially altering their contents, their ability to treat disease can be enhanced [206]. The drug-carrying modes of co-incubation, electroporation [207–209] (Fig. 4C and D), saponin treatment [210,211], sonication [212], freeze–thawing [213,214], and extrusion are described below and the success obtained with these modes is summarized in Table 1, followed by a discussion of the powerful and inspiring functionalized exosomes derived from these modes in recent years and their therapeutic efficacy.

**Table 1.** Drug-carrying modes of exosomes and their success

Method	Source	Contents	Function	Reference
Co-incubation	Exosomes from LNCaP- and PC-3 prostate cancer cells	Paclitaxel	Targeted killing of cancer cells	[80]
Electroporation	Exosomes of BM-MSC cells	Galactose lectin-9 siRNA	Triggering anti-tumor immunity	[84]
Electroporation	Exosomes of dendritic cells	GAPDH siRNA	Delivery of siRNA to the mouse brain by injection using targeted exosomes	[154]
Co-incubation	Macrophage exosomes	Adriamycin	PH response, targeted delivery, and killing of cancer cells	[168]
Co-incubation	Exosomes from HEK 293T cells	Acoustic sensitizer Chlorin e6 (Ce6) and immune adjuvant R848	Induces reprogramming of macrophages from an immunosuppressive M2-like phenotype to an antitumor M1-like phenotype, further activating effector T cells and restoring the immunosuppressive microenvironment	[173]
Electroporation	Exosomes of bEnd.3 cells	Hairpin decoy oligodeoxynucleotide (ODN) for the transcription factor RBP-J	Amelioration of non-alcoholic fatty liver disease by bone marrow-specific blockade of Notch signaling	[207]
Electroporation	Exosomes of hepatic stellate cells	Cas9 RNP	Tissue-specific gene therapy for liver disease	[208]
Electroporation	Exosomes of HUVEC cells	microRNA-29b mimic	Repairing the myocardium and preventing cardiac fibrosis after myocardial infarction	[209]
Saponin method	Milk exosome	Curcumin	Increased drug loading for the treatment of liver fibrosis	[210]
Saponin method and co-extrusion	Exosomes of HEK293 cells	Superoxide dismutase	Scavenges reactive oxygen species and reduces oxidative damage	[211]
Freeze–thaw, sonication, and co-extrusion	Milk exosome	PD-L1 inhibitors	Cancer vaccine, T cell immune surveillance, and killing of tumor cells	[212]
Freeze–thaw	Human plasma exosomes	Methotrexate	For analyzing drug metabolism in the body	[213]
Freeze–thaw	Endometrial exosomes	Human chorionic gonadotropin	Increase endometrial tolerance	[214]
Co-incubation	Exosomes of RAW 264.7 cells	Linezolid	Treatment of methicillin-resistant *Staphylococcus aureus* intracellular infections	[215]
Co-incubation	Exosomes of HEK293T cells	MicroRNA-34a	Treatment of oral squamous cell carcinoma	[216]
Electroporation	Exosomes of HEK293T cells	circRNA SCAR	Promotes macrophage polarization to M2 subtype and alleviates sepsis	[217]
Saponin method	Endothelial cells, cancer cells, stem cells, etc.	Hydrophilic porphyrin	Increased drug loading and enhanced cellular uptake of drugs	[218]

BM-MSCs, bone marrow-mesenchymal stem cells; PD-L1, programmed cell death-ligand 1; GAPDH, glyceraldehyde-3-phosphate dehydrogenase

Because the exosome membrane is similar to the cell membrane, some small molecules or lipid-soluble drugs can easily penetrate the phospholipid bilayer and enter the exosome interior. Therefore, loading such drugs can be accomplished by simple co-incubation. Yang et al. [215] successfully prepared exosomes that could be used to treat intracellular bacterial infections using a co-incubation method. Some classical anticancer drugs, such as paclitaxel [80] and doxorubicin [168], have also been loaded into specific exosomes, thus enabling targeted drug delivery for cancer and reducing the high toxicity of these drugs to a certain extent. Wang et al. pioneered the simultaneous co-incubation of sonic sensitizers and immunoadjuvants into exosomes. The local administration of anticancer drugs was completed by injection and ultrasound irradiation in the affected area, further limiting the systemic side effects of these drugs [173]. In addition to traditional drugs, some short-stranded nucleic acids can also be loaded into exosomes owing to the mild co-incubation conditions. Deng et al. [216] used cholesterol to modify microRNA-34a and enhance its fat solubility and stability and then loaded it into exosomes of HEK293T cells by co-incubation; it was successfully taken up by oral squamous cell carcinomas. Subsequent experiments demonstrated that these exosomes loaded with microRNA-34a significantly inhibited the proliferation, migration, and invasion of HN6 cells by down-regulating SATB2 expression.

Similar to the principle of new functional exosomes generated by membrane fusion, some substances originally rejected from the membrane can enter the exosome by destabilizing the original membrane of the exosome. When the influencing factors are removed, membrane stability is restored, and stable drug-carrying exosomes can be obtained; for example, Fan et al. [217] successfully loaded a steatohepatitis-associated circRNA ATP5B regulator into exosomes by instantaneously applying a certain voltage and perforating the membranes; these exosomes gained the ability to promote the polarization of macrophages toward the M2 subtype. Similarly, Fuhrmann et al. [218] successfully loaded drugs into exosomes of different origins using electroporation. Apart from the electroporation method, they also attempted various other loading methods, such as the extrusion and saponin methods, and evaluated the drug-loading capacity of these methods. The saponin-assisted method resulted in an 11-fold increase in hydrophilic porphyrin loading. The optimal drug-loading method differs for different exosomes and the drugs they load. Therefore, a side-by-side comparison is necessary for subsequent production.

Gunassekaran et al. [219] employed both exogenous and endogenous loading to successfully produce a highly targeted and efficient cancer vaccine. Using tumor-associated macrophages (TAMs) as an entry point, they first transfected M1 exosomes with NF-κB p50 siRNA and miR-511-3p to promote M1 polarization and then surface-modified the genetically engineered exosomes with the IL4R-binding peptide IL4RPep-1 to target the IL4 receptor in TAMs. These exosomes can reprogram TAMs into M1-like macrophages, resulting in effective tumor suppression.

##### Endogenous loading

The endogenous loading method aims to induce mother cells to secrete the relevant therapeutic factors. These therapeutically relevant factors are often not autonomously produced by the mother cells. Genetic engineering is one of the most prominent approaches to achieve endogenous production of therapeutically relevant substances and plays a role in the prevention and treatment of various diseases. Bu et al. [49] loaded IL-10 mRNA modified with miR-155 into macrophages; when this miR-155 was forced to be expressed, the translational program of IL-10 mRNA was activated, and a large amount of the anti-inflammatory factor IL-10 was expressed intracellularly. These products were encapsulated and excreted in exosomes and alleviated atherosclerosis in mice. Zhang et al. [220] successfully engineered liver cells by intravenous injection of neuron-targeting rabies virus glycoprotein and mutant huntingtin siRNA. These engineered cells produced exosomes with marked therapeutic effects against neurodegenerative diseases.

Genetically engineered modified mother cells can maintain stable secretion for a certain number of generations, thus circumventing the tedious steps in repeated modifications and stringent requirements for operators and instruments. In addition, the specific modification of exosomes by genetic engineering technology is a stable, reliable, and convenient process, and further purification of the exosomes is not required; thus, the quality of the product is ensured. However, whether the loaded genes can be stably expressed according to researchers’ expectations remains unknown, and constant testing is needed, which undoubtedly increases the cost of this method in the research and development stages. The state of the cells can also directly affect the quality and yield of exosomes; to obtain high-quality exosomes, superior cell culture conditions are required, which in turn casts a shadow over the widespread use of this technology in loading drugs.

#### Summary

Strategies for enhancing exosome functionality primarily focus on 3 core directions: expanding sources, exogenous loading, and endogenous loading. These approaches aim to overcome inherent limitations in natural exosomes regarding targeting, drug-carrying capacity, and therapeutic efficacy, each offering distinct advantages, application scenarios, and challenges to be addressed.

The core value of expanding exosome sources lies in discovering novel vesicles with unique biological functions. This strategy breaks away from the traditional reliance on human immune cells or stem cell-derived exosomes, broadening the research scope to encompass a wider range of physiological and pathological microenvironments. This approach significantly enriches the resource pool of “natural carriers” and “active components” for therapeutic applications. Particularly noteworthy is the emergence of plant-derived exosome-like nanoparticles (PENs). Leveraging their abundant sources, low immunogenicity, and innate ability to cross biological barriers, PENs demonstrate immense potential in tumor-targeted therapies and indirectly modulating systemic metabolism by “training” the gut microbiota. These novel exosome sources, with their unique membrane protein markers and specific internal nucleic acid payloads, serve as valuable functional modules for subsequent engineering modifications. However, expanding sources also introduces significant risks and uncertainties. The primary concern is biosafety. The immunogenicity of heterologous exosomes is difficult to predict, potentially triggering clearance responses or hypersensitivity reactions that reduce efficacy or cause adverse effects. Secondly, clarity of the mechanism of action remains a critical bottleneck. The specific targets and signaling pathways of many newly discovered exosomes are not yet fully elucidated. For example, the precise molecular mechanisms by which cancer cell exosomes promote angiogenesis are complex and may carry carcinogenic risks. Finally, the feasibility of large-scale production poses a practical barrier to translational application. Efficiently, stably, and cost-effectively isolating high-purity target exosomes from complex biological matrices remains a major technical challenge.

Exogenous loading methods aim to flexibly confer therapeutic functions. Their greatest advantage lies in flexibility and universality. Co-incubation methods offer mild conditions suitable for lipophilic small molecules or modified nucleic acids, while methods like electroporation, sonication, and saponin treatment overcome membrane barriers to achieve efficient loading of hydrophilic macromolecules such as proteins and certain nucleic acids. This enables researchers to select “weapons” and load them into “vehicles” with near-unrestricted flexibility based on disease requirements [221]. Comparative studies across multiple methods provide valuable data for selecting optimal processes for specific “carrier-drug” pairings.

The primary drawback of exogenous loading methods lies in their potential to compromise the natural integrity and function of exosomes. Aggressive physicochemical treatments may disrupt the integrity of the exosomal membrane, leading to leakage of contents, denaturation of surface proteins, and consequently affecting their inherent targeting capabilities and stability during in vivo circulation. Controlling drug loading efficiency and encapsulation rate presents another major challenge: insufficient loading yields inadequate therapeutic effects, while excessive loading may compromise the stability of the carrier itself. Furthermore, ensuring uniformity of drug-loaded exosomes is difficult, with batch-to-batch variations potentially compromising the reproducibility of therapeutic outcomes. Thus, while powerful, this method requires a delicate balance between loading efficiency, carrier integrity, and process stability.

The endogenous loading approach directly modifies parent cells through techniques like genetic engineering, enabling them to encapsulate target therapeutic molecules during exosome production—effectively conferring function at the source. The core advantage of this strategy lies in the naturalness and homogeneity of the resulting product. By genetically engineering progenitor cells to stably express target proteins or nucleic acids, these molecules are subsequently incorporated into exosomes via the cells’ own biosynthetic pathways. This approach maximally preserves the natural structure and secretion mechanism of exosomes, yielding products with intact membrane structures, stable loading, and relatively good batch consistency. This approach avoids cumbersome post-loading processes and theoretically yields functionalized exosomes closer to their natural state.

The primary challenges facing endogenous loading methods are technical complexity and cost. Establishing stable gene transduction and expression systems requires extensive molecular biology expertise and is both time-consuming and labor-intensive. Uncertainty in cell condition poses another marked risk. Genetic modification may disrupt the parent cells’ normal physiology and proliferation capacity, thereby affecting exosome yield, composition, and even safety. Consequently, while promising, this approach remains costly during research and development, demands extremely stringent cell culture conditions, and is thus limited in large-scale production and widespread application.

In summary, the 3 major strategies—expanding sources, exogenous loading, and endogenous loading—collectively form the technological landscape for enhancing exosome functionality. These approaches are not mutually exclusive but exhibit complementary and convergent trends: exosomes from novel sources serve as excellent natural carriers or functional module libraries; exogenous loading offers flexible and rapid options, while endogenous loading focuses on achieving more stable engineered products.

## Application of Enhanced Exosomes

In addition to the improved therapeutic ability and diagnostic accuracy of engineered exosomes compared with the natural exosomes, these functionalized and enhanced exosomes can be combined with other therapeutic tools, such as magnetic fields and PTT, thus playing a stronger role in these areas. Structurally modified exosomes can also be involved in other ancillary functions such as bioimaging.

### CNS-targeted therapies that penetrate the BBB

Exosomes can easily cross most barrier systems in the body because of their specialized membrane structures. However, exosomes do not perform well when facing the BBB. In the past, researchers have found that exosomes produced by some cancer cells have a strong ability to penetrate the BBB. The functional modification of exosomes induced by these cells is a proven solution to the difficulty faced by commonly used natural exosomes in penetrating the BBB. In 2019, Morad et al. [222] elucidated a specific strategy to enable the crossing of the BBB by some cancer cell-derived exosomes; these tumor-derived exosomes could circumvent the low physiological transcytosis rate under physiological conditions by decreasing the cerebral endothelial expression of rab7 and increasing its transport efficiency. Cheng et al. [223] used this approach as the basis for developing nanoreactors. In this study, brain metastatic breast cancer cell-derived exosomes provided membranes with high BBB permeability, and internally encapsulated reactive manganese ions, arsenate, and glucose oxidase activated the tumor suppressor gene, P53, to cut off glucose supply. Fang et al. [224] utilized the high BBB penetration of glioblastoma exosomes and successfully produced a function-enhancing exosome by filling the exosome with a variety of active ingredients. Among them, Mn, Bi_2_Se_3_, and other components convert hydrogen peroxide into hydroxyl and superoxide radicals under NIR-II light irradiation, thus resulting in highly targeted and potent killing of glioblastoma.

When confronted with organic pathologies, immune cells generate various types of responses, attracting researchers because of their ability to target diseases and penetrate barriers. In addition to the fact that these cells respond to almost all pathologies produced by the organism, it is logical that they and the exosomes they produce are the focus of research in this area. Wang et al. [225] utilized the strong migratory properties of neutrophils and their strong affinity for inflammatory sites to achieve precise inhibition of glioblastoma by fusing neutrophil membranes to exosomes as a means for transporting adriamycin, which has difficulty crossing the BBB, to the site of the disease in a smooth and targeted manner. Tao et al. [226] used exosomes produced by natural killer cells as the raw material, modified their surfaces with antigens, and simultaneously loaded drugs that disrupted the iron death defense mechanism inside the exosomes to achieve targeted cancer therapy that penetrated the BBB. Specifically, the transferrin receptor-binding peptide on the exosome surface was modified to allow the exosome to pass through the BBB, and the chemotaxis of natural killer cells in cancer transported the exosome to the affected area. The temporal and spatial accuracies of drug release can be further enhanced by combining reactive oxygen species (ROS) amplification with PDT. Functionally enhanced exosomes designed in this manner can be systemically injected to produce a highly targeted effect. It can effectively inhibit cancer while significantly minimizing the side effects of drugs. In addition to modifying the exosome itself, stimulation of the mother cell using physical methods can also alter the cell physiology and thus enhance the exosome function. Deng et al. [227] designed a novel exosome that could reverse oligoamyloid β-induced cytotoxicity to alleviate Alzheimer’s disease in vitro by ultrasonically treating astrocytes. Subsequently, they used focused ultrasound with microbubbles to open the BBB to deliver the exosome into the BBB and facilitate in vivo clearance of amyloid β plaques. Some plants produce EVs that have the ability to cross the BBB along with certain therapeutic functions. However, the underlying mechanism remains unclear, and the specific process of crossing the barrier must be investigated for practical use.

### Cancer vaccines

Conventional cancer treatments have shortcomings such as nonspecific killing and numerous serious side effects. Novel immunotherapies based on the immune system have been developed recently to solve these problems. Exosomes, as multifunctional nanoparticles, have attracted much attention because they can load various molecules on the surface or inside, in cases where heterologous immunity is weak, and have obtained favorable results.

#### Activation of relevant immune cells

**Fig. 5.**
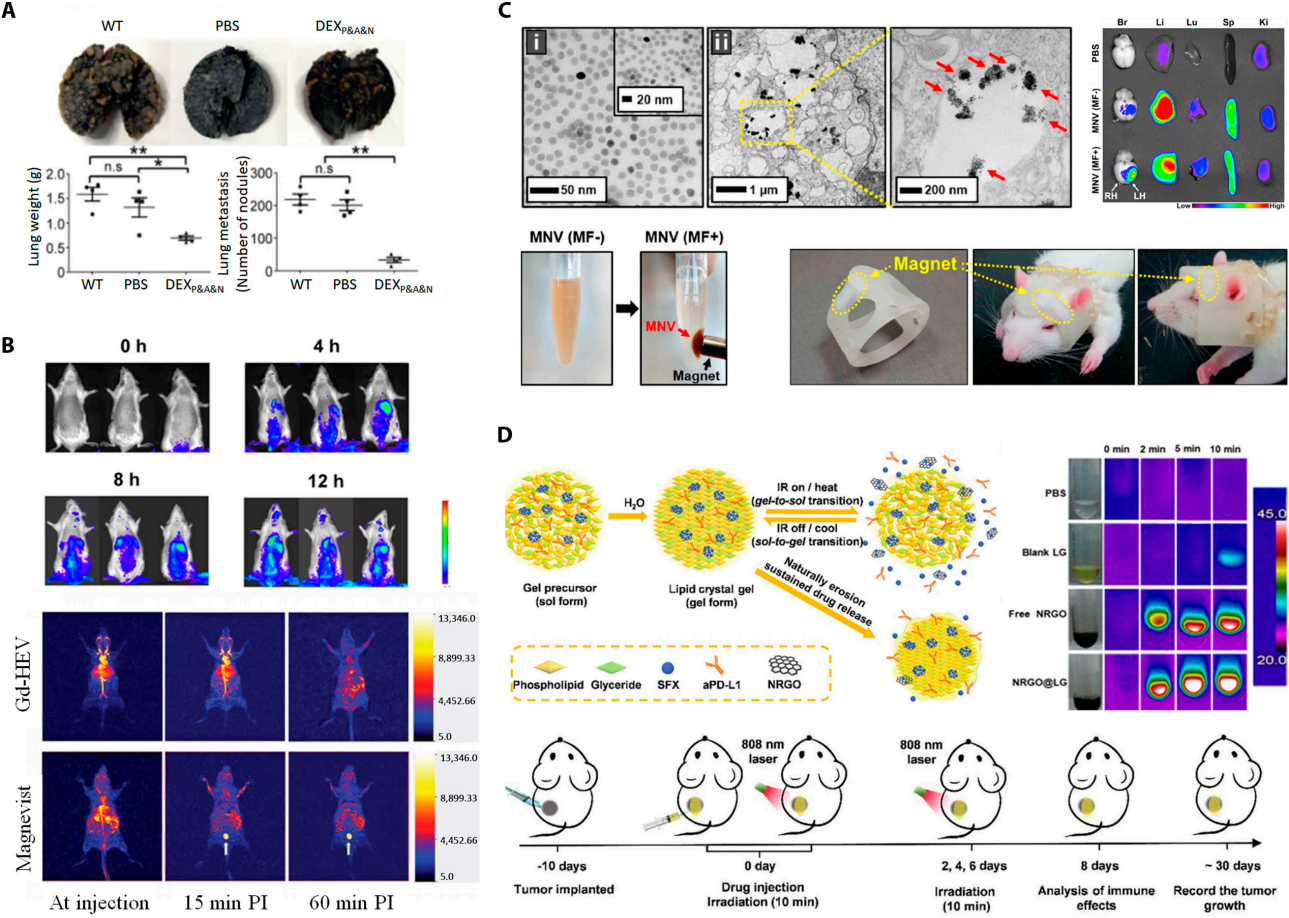
Applications of enhanced exosomes. A) Exosomes loaded with immune substances can be used as a new type of highly targeted cancer vaccine for cancer immunotherapy by reversing the tumor microenvironment or activating cellular immunity. Reproduced under terms of the CC-BY license. [228] Copyright 2022, Springer Nature. B) After a simple loading process, the exosomes can be prompted to carry substances with good fluorescence or thermal imaging response, thus providing a new means for bio-imaging. Reproduced under terms of the CC-BY license. [245] Copyright 2023, Springer Nature. Reproduced with permission. [246] Copyright 2020, Royal society of chemistry. C) Iron oxide nanoparticle (IONP) exosomes can be induced by a magnetic field to concentrate in large quantities to a designated site, and then by means of ultrasonic crushing, high concentration drug delivery can be realized to the brain and other sites with a barrier effect, as shown in TEM images comparing (i) bare iron oxide nanoparticles (IONPs) and (ii) exosomes loaded with IONPs. Reproduced with permission. [258] Copyright 2020, Elsevier Ltd. D) Exosomes loaded with photothermal agents obtained by exogenous loading and surface modification can enable highly targeted PTT. Reproduced under terms of the CC-BY license. [267] Copyright 2024, Wiley-VCH.

The immune system is activated as a result of the interaction among various immune cells, and the activation of these cells is the most direct means of treating cancer by immune means. Among these, DCs play a crucial role as important antigen-presenting cells. Simple targeted modification of the exosome surface is a common strategy [228] (Fig. 5A). Huang et al. [85] attempted to load human neutrophil elastase and hiltonol into a breast cancer-derived exosome. This exosome activated type 1 conventional DC (cDC1) in situ and activated CD8^+^ T cell responses in a mouse xenograft model of triple-negative breast cancer and in patient-derived tumor-like organs, demonstrating excellent tumor suppressor capacity. Wang et al. [87] proposed a simpler protocol, in which they co-incubated the collected induced pluripotent stem cell-derived exosomes with DCs and simultaneously administered pulsed stimulation to obtain a cancer cell vaccine that could effectively activate T cells both inside and outside the vector. Follow-up experiments demonstrated that the vaccine maintained long-term T cell responses in mice and effectively inhibited melanoma growth and lung metastasis.

T cells are important executors of anticancer cellular immunity, and the development of memory CD8(+) cytotoxic T lymphocytes is key to antitumor immunity. In a 2012 study, Xie et al. [229] succeeded in designing an exosomal vaccine that stimulated cytotoxic T lymphocyte responses through IL-2 and co-stimulatory CD80 signaling. Subsequent studies revealed that the interaction between the co-stimulatory molecule OX40 and its ligand OX40L produced key signals for T cell activation, and Cheng et al. [230] successfully obtained an exosome vaccine expressing programmed death 1 (PD-1) and OX40 ligands by genetically engineering exosomes. The exosomes were also embedded in monoclonal antibodies specific for human T cells CD3 and epidermal growth factor receptor (EGFR), which could activate T cells and achieve effective killing and long-term immunization of EGFR-positive triple-negative breast cancer cells in an in vivo model.

Enhancing the immunogenicity of exosomes can further improve the therapeutic outcomes. Zhang et al. [231] attempted to load a variety of peptide neoantigens onto the surface of serum exosomes and obtained an exosome vaccine capable of promoting lymph node homing and DC uptake in a long-term and effective manner, and subsequent experiments demonstrated that the vaccine had an inhibitory effect on cancer such as colon carcinoma and melanoma. The vaccine not only inhibited tumor growth and stimulated immune responses in 3-dimensionally cultured colon cancer microenvironments but also achieved tumor eradication and long-term immune memory in conjunction with a PD-1 antibody in a mouse model of colon cancer. Li et al. [232] adopted a similar idea to produce a batch of exosomal vaccines containing novel antibodies. The difference is that they used exosomes produced by DCs as the raw material, and thus, the exosomes were able to trigger stronger antigen-specific broad-spectrum T-cell- and B-cell-mediated immune responses while having better biocompatibility. In contrast, Wang et al. [233] derived macrophage–tumor hybrid cells that could directly secrete exosomes with immunogenic properties by using mother cells as a starting point, extracting nuclei from tumor cells, and transplanting them into activated M1 cells. The exosome vaccine obtained in this way induced immune stimulation through classical antigen-presenting cells; it can be spontaneously enriched toward lymph nodes as well as tumorigenic sites upon injection and has demonstrated effective inhibition and long-term immune effects in a variety of mouse models of cancer, including lymphoma, mammary carcinoma, and melanoma. This strategy of generating exosome vaccines from hybridized cells eliminates processing costs and prevents exosome structural damage and batch-to-batch variation during production, further stabilizing the vaccine quality. This method has the potential for large-scale production.

#### Altering the TME to inhibit immune escape

The environment surrounding the tumor is also somewhat altered by cancer cells and their associated cells, resulting in pH abnormalities, hypoxic environments, abnormal vascular proliferation, remodeling of stromal components, and changes in the composition and content of associated cytokines. These changes often cause the immune system to be highly suppressed in the TME, and the relevant cells can help cancer cells to achieve immune evasion, resulting in the failure of cancer vaccines to achieve the desired effect. Therefore, the TME needs to be adjusted.

Many immune cells exist in the tumor environment, among which TAMs play an important role. TAMs act as macrophages in the TME. However, this “messenger” often tampers with the signals it receives, helping tumor cells evade the immune system. PD-L1 molecules of this class of cells are entwined with PD-1 receptors on CD4^+^ and CD8^+^ effector T cells, and T cell suppression is achieved by inhibiting T cell receptor signaling [234]. In addition, at primary sites, TAMs promote tumor cell invasion and tumor stem cell viability, while inducing angiogenesis. At metastatic sites, TAMs maintain tumor cell dormancy in some cases [235].

Therefore, targeting TAMs is key to reversing immunosuppression in the TME. Chen et al. [236] developed an engineered milk exosome vaccine modified with an M2 macrophage-binding peptide and anti-EGFR nanobodies to specifically deliver siPD-L1 into M2 macrophages, thereby repolarizing them to M1 macrophages. In the TME, the proportion of M2 macrophages was up-regulated and macrophage-mediated innate immunity was suppressed. Yu et al. [237] focused on the interconversion of M2 and M1 cells, where engineered M1 macrophage exosomes activated adaptive immunity by activating the OX40/OX40L pathway to enable reprogramming of M2-like TAMs into M1-like macrophages for therapeutic purposes.

In addition to M2 macrophages, cancer-associated fibroblasts (CAFs) play an important role in constructing the cancer microenvironment. These cancer cells acquire drug resistance by secreting various bioactive substances, including exosomes and nucleic acids, and inhibiting iron death [238]. They can also promote tumor development by enhancing cellular nutrient uptake and flow [239]. Freag et al. [240] elucidated the process by which cancer cells convert normal cells into CAF: first, cancer cells convert normal fibroblasts into CAF by secreting exosomes; then, the cancer cells activate the relevant cellular pathways to maintain CAF survival. Freag et al. proposed that effective inhibition of these 2 links could prevent the production of CAF, thereby disrupting the cancer microenvironment. Subsequently, the team successfully fabricated targeted nanoliposomes to block both nodes in the fibroblast growth factor receptor pathway and demonstrated their excellent immune-activating ability in subsequent experiments.

Setua et al. [241] found that stromal cells derived from normal tissues adjacent to pancreatic tumors secreted tumor-targeted exosomes while maintaining normal morphology and physiological functions. This finding holds promise for the development of exosome-based vaccines that maintain high immune activation capacity in the TME. Inspired by this, Yuan et al. [242] transformed the CAFs surrounding the exterior of the tumor into an enclosure loaded with antitumor drugs. Specifically, drugs were delivered into the CAFs by preparing a lipid–polymer hybrid drug delivery system, and the transformed CAFs effectively killed cancer cells by secreting drug-loaded exosomes. This scheme effectively alleviates the problem of immunosuppression in the cancer microenvironment and provides a direction for the innovative application of enhanced exosomes, that is, using peripheral cells as a jumping-off point, thus generating functionally enhanced exosomes in the in vivo environment. The low PH and ROS generation triggered by the hypoxic state of the TME can also be used as a trigger for developing relatively responsive therapeutic regimens for different conditions in the TME [243].

### Bioimaging

Various enhanced exosomes with strong targeting properties have been described previously, and these powerful nanoparticles can deliver content to a target site with precision. The delivered content can be limited to drugs as well as various contrast agents, probes, or fluorescent dyes, ensuring high accuracy in bioimaging. Simultaneously, the more stable physicochemical environment inside the exosome ensures that these reagents with delicate structures avoid the fate of decomposition or destruction. Yang et al. [244] synthesized a novel probe that can be used for single-molecule localization microscopy by using hydrophilic cesium lead bromide chalcogenide nanocrystals and PEG and successfully loaded it into exosomes with a diameter of <150 nm.

After years of development, exosomes carrying various probes or dyes have shown satisfactory results in various classical bioimaging fields such as magnetic resonance imaging (MRI) [245–247] (Fig. 5B), radiography, computed tomography [248,249], nuclear medicine imaging [250], and fluorescence optical imaging [251]. Magnetic particle imaging is a novel imaging technique that overcomes the long-standing limitation of MRI of providing only contrast by detecting superparamagnetic iron oxide (SPIO) nanoparticles based on the tracer [252]. It is also less harmful and safer for patients than radiography [253]. Jung et al. [254] isolated 4 exosomes from differently treated human breast cancer cells and selected the one with the strongest affinity for hypoxic cancer cells using fluorescent lipophilic tracer 3,3′-dioctadecyloxacarbocyanine perchlorate labeling and loaded SPIO nanoparticles and olaparib, successfully constructing a diagnostic and therapeutic integrated exosome drug delivery strategy to support magnetic particle imaging technology.

The process of spontaneously converting some structurally simple compounds into complex substances with the ability to visualize them without human intervention has attracted much attention. Qambrani et al. [255] designed a silver DNA nanocluster using biosynthesis, encapsulated in cancer cell exosomes, and achieved high-precision targeted delivery by exploiting the homing properties of cancer cell exosomes. Tayyaba et al. [256] cultured HepG2 cells in a glutathione-containing environment that reassembled ions from AgNO_3_ and FeCl_2_ into nanoclusters with both fluorescent probe capability and contrast agent potential. Surprisingly, these nanoclusters can be loaded into exosomes to achieve targeted contrast in cancer cells such as HepG2 and U87 cells. This protocol does not require manual isolation and processing of exosomes, thus ensuring exosome integrity and saving production costs, suggesting the potential for large-scale, high-quality production.

### Magnetic field induction

Currently, most exosomes are administered by injection, a delivery method that relies heavily on the choice of injection location and targeting of the lesion area by the exosome. Magnetic field induction provides a new perspective on treatment options. By directly loading magnetic substances, such as SPIO nanoparticles onto exosomes, magnetic field induction can be performed, which can be targeted and driven at the macroscopic level [257]. Kim et al. [258] found that iron oxide nanoparticles stimulate MSCs to produce exosomes containing high concentrations of therapeutic factors (Fig. 5C). They improved the locomotor ability of ischemic stroke model rats by systemically injecting exosomes produced by MSCs treated with iron oxide nanoparticles and guided by a magnetic field. This treatment strategy increased the accuracy of localization of ischemic lesions by 5.1-fold compared with a single injection. Liu et al. [259] designed a magnetically targeted exosome therapeutic system with PH responsiveness on this basis. Specifically, exosomes with therapeutic functions were connected to a magnetic shell containing Fe_3_O_4_ via acylhydrazone bonds and antibodies, and under the guidance of a magnetic field, these particles were aggregated toward the damaged tissues to achieve the release of exosomes under the acidic conditions of the damaged tissues. Li et al. [260] designed an enhanced exosome that had both penetrating and targeting capabilities; exosomes loaded with Fe_3_O_4_ nanoparticles could penetrate the BBB and be enriched toward the brain under localized magnetic localization. Villa et al. [261] used a different approach by loading surface-modified exosomes with therapeutic ability onto ferromagnetic nanotubes and successfully delivered the exosomes to the affected area using an external magnetic field; using this approach, they reversed Duchenne muscular dystrophy in mice.

### PTT, infrared targeting, and ultrasound-targeted destruction

Multiple clinical cases have proven that it is difficult to effectively treat a disease by relying on only one treatment; the current strategy is to use multiple independent treatments concurrently, utilizing multiple mechanisms to alleviate the symptoms of a said disease or eradicate it. Therefore, novel and effective therapeutic methods are urgently needed for clinical treatment. PTT, infrared targeting, and ultrasound-targeted destruction have been the most commonly used adjuvant therapeutic methods in recent years; however, these methods have certain requirements for drug targeting, requiring the drug to be enriched at a specified location before the operation can be performed. Natural exosomes often do not carry the various response reagents required to engage these therapeutic modalities. Various targeting-enhanced exosomes can solve this problem by bringing the drug to the affected area while maintaining the stability of the drug’s physicochemical properties.

PDT, which mainly utilizes ROS generated by photosensitizers to treat cancer, is a classical noninvasive therapy. In a study by Cheng et al. [262], chimeric peptide-engineered exosomes demonstrated excellent suitability of PDT. The treatment is achieved by a 2-step response of the exosome to the radiation signal, where the first step triggers photochemical internalization and lysosomal escape from the cell, which can promote the uptake of exosomes by the target cell. The second step triggers the reaction of the photosensitizer to generate large amounts of ROS that destroy the cancer cell nucleus. Under the influence of PDT, cancer cells produce exosomes that induce DC maturation and activate the immune system, thus participating in cancer immunotherapy [263]. Guo et al. [264] demonstrated the effect of a combined approach involving PDT and immunotherapy. In this system, exosomes containing PD-L1 monoclonal antibodies play a targeted delivery role and drive a T cell-directed immune response with controlled release of the antibodies in an acidic TME. Subsequently, the embedded photosensitizers, indocyanine green, and activated manganese dioxide, produce large amounts of ROS in a radiation-induced manner. Mn^2+^ generated by this process also reverses TAMs, reestablishes the immune microenvironment, and activates T cells.

Although PDT is minimally invasive and has low toxicity, the ROS produced by this method can still be toxic to the surrounding normal tissues, especially when applied to intracranial tumors, and damage to the positively yielding cells may be unacceptable. Therefore, high-performance and low-toxicity photosensitizers are required.

Novel photosensitizers for PDT have been developed in recent years. Deiana et al. fabricated a novel photosensitizer that targeted cancer cell nucleic acids and induced genomic destabilization. More importantly, this photosensitizer was preferentially localized in ILVs, which might provide ideas for the subsequent convenient production of exosomes containing photosensitizers [265]. In contrast, photosensitizers based on tripodal quinone-anthocyanine dyes developed by Muramoto and Sakamoto [266] are more focused on targeted cancer selectivity and are capable of producing high targeting selectivity for guanine-quadruplexes. With the support of these high-performance photosensitizers, PDT may have an increased scope for action.

Unlike PDT, which kills cells by generating ROS, PTT utilizes the high heat generated by a photothermal agent when it is exposed to radiation to ablate tumor cells and release drugs [267] (Fig. 5D). Liu et al. [268] successfully loaded photothermal agents inside exosomes using a simple electroporation method to achieve efficient in vivo tumor ablation. Single PTT is more effective for solid tumors but does not completely kill all tumor cells. Huang et al. [269] proposed a combined photothermal and chemotherapeutic treatment by loading the photothermal agent IR780 and the anticancer drug lenvatinib simultaneously into the interior of exosomes. In a detailed study, by employing serum exosomes from tumor-bearing mice with high affinity for tumor cells as raw materials, Liu et al. [270] developed a targeted photothermal therapeutic tool and combined it with immunotherapy. The experimental results demonstrated that this therapeutic regimen was able to significantly ablate tumors while promoting T-lymphocyte infiltration into tumor tissue, which is welcome news not only for PTT but also for the expanded use of immunotherapy because despite the high targeting of exosomes in vivo, the complex immune environment in the diseased region, especially in the TME, where CAFs and other related cells are present, makes it difficult for immunotherapy to achieve the predicted results. Therefore, the combination of immunotherapy and PTT can compensate for poor penetration and low prognostic ability of photosensitizers, respectively, and is of great significance.

Despite the similarities in the treatment process, PDT and PTT are 2 separate therapies in terms of their principles of action. Thus, the toxicity of the 2 treatment options is not superimposed, but the therapeutic capacity is significantly increased. Combination therapies are gaining momentum and have been shown to be effective [271]. However, the number of relevant therapeutic cases remains small, and strategies for the production and design of exosomes required for combination therapy need to be further investigated. In short, addressing the limitations of each modality and improving the therapeutic safety and efficacy of combination therapies are the challenges of this therapeutic strategy that must be addressed.

Ultrasound-targeted microbubble disruption is a direct drug release method. This method is powered by a low-frequency, medium-power ultrasound, which triggers exosome cavitation at a designated site, thereby enabling targeted drug release [272]. However, the effect of exosome induction is not obvious by itself, and highly targeted exosomes are required to achieve therapeutic expectations. Sun et al. [273] encapsulated engineered mRNAs into exosomes and activated them by ultrasound in specific adipose tissues. Chen et al. [274] inhibited adriamycin-induced heart disease by targeting engineered exosomes carrying siRNA for the destruction of the heart. The ultrasound-targeted microbubble disruption technique has a low impact on normal cells, releases drugs at refractory tissue sites that are not easily receptive to exosomes, such as the heart, adipose tissue, and skeletal muscle [275], and is suitable for combined therapy with a variety of other therapeutic modalities [276]. Some studies have shown that when performing ultrasound disruption of microbubbles, the resulting mechanical and cavitation effects can increase the permeability of cell membranes, widen the endothelial cell gap [277], and enhance the uptake of drugs released from exosomes in the target cells; this enhanced uptake property makes ultrasound-targeted delivery a highly promising delivery modality. However, relevant studies are still scarce, and it is worthwhile to continue exploring this field.

## Conclusion

Exosomes have proven to be an effective tool for the diagnosis and treatment of diseases in the past decades and have been strongly emphasized in current literature, becoming a hot topic in the field of nanomedicine therapy. However, the inefficiency and high cost of traditional separation techniques are major obstacles to the wider application of exosomes. The maturity of aptamers and microfluidic technologies has led to the hope of overcoming this dilemma. On the one hand, these technologies have given rise to several highly efficient separation techniques based on the principles of immunoaffinity and volume grouping by combining them with the principles of traditional separation methods. On the other hand, microfluidic chips have also fully utilized their advantages to develop new separation techniques based on new principles such as electrophoresis and acoustic force, which have largely expanded the means of separation and given operators more choices. The exosomes obtained using these new techniques have higher integrity and activity than those obtained using traditional methods [278]. However, these technologies often require personalized design according to the characteristics of exosomes, such as a specific microfluidic chip microstructure. Researchers may need to prepare a variety of chips to cope with several different types of exosomes such that production efficiency and cost cannot be well confronted with new types of exosomes that will continue to appear in the future. Therefore, reducing the production cost of personalized designs may be an urgent problem for these new technologies.

However, each separation technique inevitably has some problems owing to inherent defects in its separation principle. For example, the Joule heat generated by electrophoresis-based separation techniques may reduce the activity of the exosomes obtained, and exosomes separated on the basis of immunoaffinity may still be mixed with nontarget exosomes. The use of multiple separation methods in the past and improvements being made in separation efficiency indicate that this problem can be solved by designing integrated microfluidic chips that contain multiple separation methods. Therefore, the design and production of complex microfluidic chips that integrate multiple separation techniques could be the focus of future developments in exosome separation technology.

In addition, natural exosomes and traditional therapeutic methods alone can no longer meet current needs, and their greater potential is yet to be realized. Engineered exosomes generated using various enhancement technologies have become a breakthrough in overcoming the current situation. Exosomes obtained through genetic engineering, chemical modification, or membrane fusion can be loaded with different drugs according to the requirements of the researchers or can be included in highly targeted therapies by altering the type of surface receptors and the composition of the structure. These new exosomes can be combined with magnetic field induction, PTT, ultrasound-targeted destruction, and other methods to control and treat diseases through a variety of mechanisms. None of these methods can be realized using natural exosomes.

Although the new generation of enhanced exosomes has made up for many deficiencies in natural exosomes through the unremitting efforts of researchers, there is still room for progress in areas such as the leakage of inclusions generated during membrane fusion, difficulty of completely removing by-products and raw materials from chemically modified exosomes, and inefficiency of the mildest and simplest co-incubation technique. With the advancements in exosome research, new problems have emerged. Recent studies have shown that even exosomes of the same type have different subgroups that may play different roles in an organism [102]. However, most of the commonly used exosomes are directly extracted from animal body fluids or cellular environments, meaning most of the experiments carried out nowadays are focused on a certain type of exosome or exosomes produced by a certain type of cell; this finding does not accurately reflect the dynamics of physiology or pathology, and the results obtained are not in line with the reality. Future research will shift its focus from the simple and generalized application of “exosome mixtures” to their in-depth analysis and precise manipulation. With advancements in microfluidic and aptamer technologies, high-purity subpopulation sorting based on specific surface molecular profiles will become feasible. This may lead to the creation of “exosome subtype databases”, laying the foundation for identifying the unique physiological and pathological functions of different subtypes and enabling targeted interventions. Simultaneously, research on novel exosome sources such as skeletal muscle and plant-derived exosomes will progress from observational studies to elucidating molecular mechanisms. This includes investigating their ability to traverse specialized biological barriers and their interactive networks with microenvironments like the gut microbiota, thereby safely and efficiently translating their inherent therapeutic advantages. Personalized therapy and intelligent response systems may also emerge as key future development areas. The inherent low immunogenicity, excellent biocompatibility, and modifiable properties of exosomes make them an ideal platform for personalized medicine. Exosomes derived from a patient’s own immune cells or stem cells can be used to construct fully individualized therapeutic vaccines or gene drugs, minimizing rejection reactions. Supported by various editing technologies, engineered exosomes will evolve into “smart nanomachines” capable of environmental sensing and programmed responses. Primary response directions encompass 2 aspects: First, multi-stimulus-responsive drug controlled release. Designing exosome membranes or internal structures that simultaneously respond to multiple TME characteristics—such as weak acidity, high ROS, or specific enzymes—enables precise, on-demand drug release at the lesion site, minimizing off-target toxicity. Second is externally controlled physical field-guided integrated diagnosis and therapy. By combining exosomes with diverse physical fields (near-infrared light, ultrasound, and magnetic fields), we develop “diagnosis-therapy integrated” platforms capable of both high-resolution imaging and precise treatment. For instance, ultrasound-guided exosomes loaded with sonosensitizers generate localized ROS to kill tumors, while ultrasound imaging simultaneously monitors drug distribution.

Overall, the emergence of a new generation of separation and exosome enhancement technologies has addressed the problems of traditional exosomes, has broken the bottleneck of exosome application, and has become the technological basis for the application of exosomes in a wide range of scenarios. However, new challenges are still emerging, and more detailed and in-depth research is required to address the challenges in current applications of exosomes.

## Data Availability

No data was used for the research described in the article.
