# Supplementary material for: Exosome Augmentation Technologies for Drug Delivery and Disease Treatment: A Review
Source: Biomater Res. 2026 Feb 19;30:0318. doi: 10.34133/bmr.0318 (PMC12917129; doi:10.34133/bmr.0318)
Supplement: Supplementary 1 — Figs. S1 to S3 Table S1 [file bmr.0318.f1.zip › bmr.0318.f1.docx]

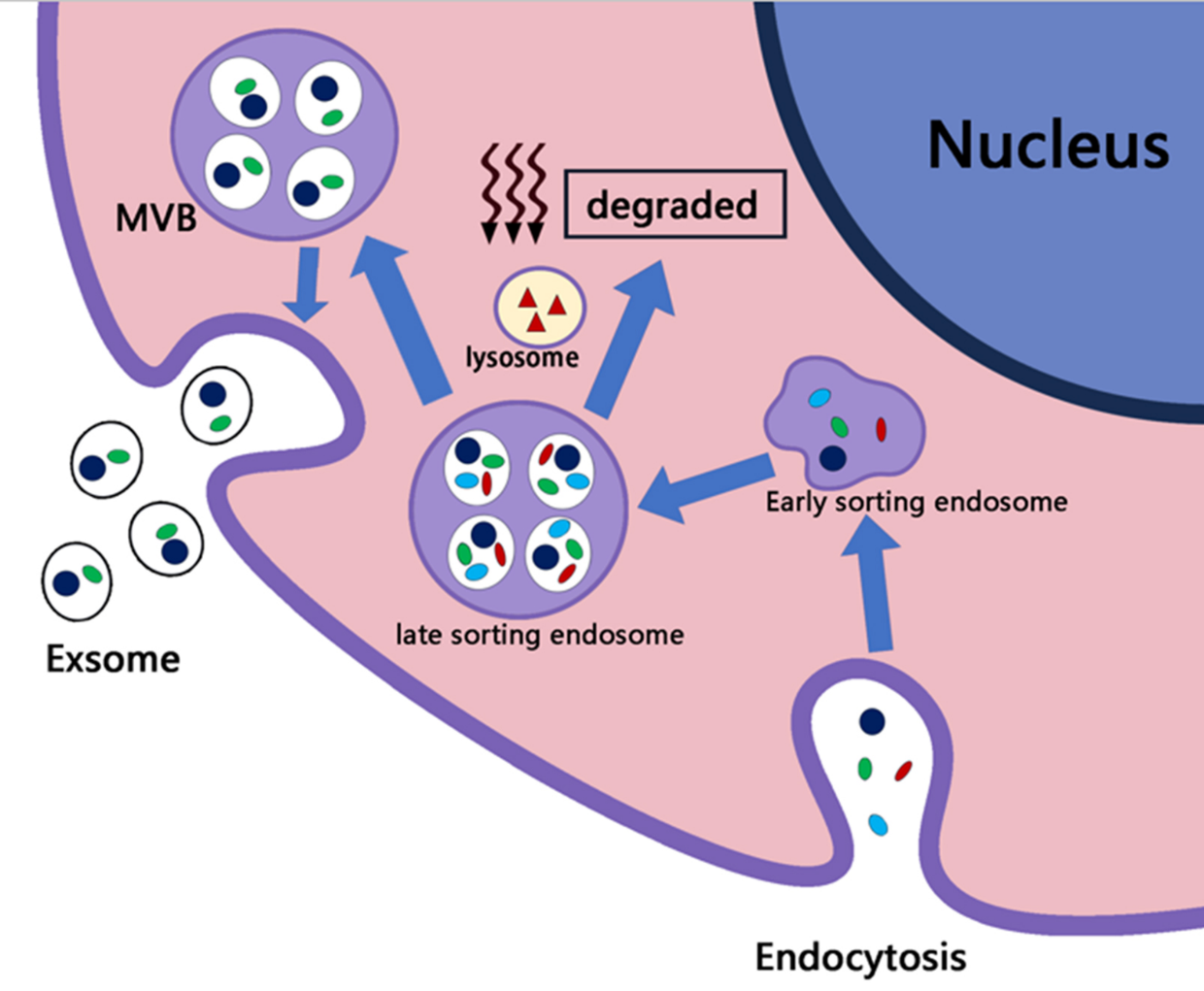


Fig S1. The Process of Exosome Formation. The formation of exosomes begins with the invagination of the cell membrane to form early sorting endosomes. These early sorting endosomes gradually mature into late sorting endosomes, whose inner membranes bud inward to form multiple intraluminal vesicles, thereby transforming into multivesicular bodies (MVBs). Subsequently, multivesicular bodies face two fates: some fuse with lysosomes for degradation, while others migrate toward the plasma membrane and fuse with it, ultimately releasing the intraluminal vesicles as “exosomes” into the extracellular space, completing their generation and secretion process.

**Fig S2. Common separation techniques for exosomes** A) Successive ultracentrifugation steps to remove
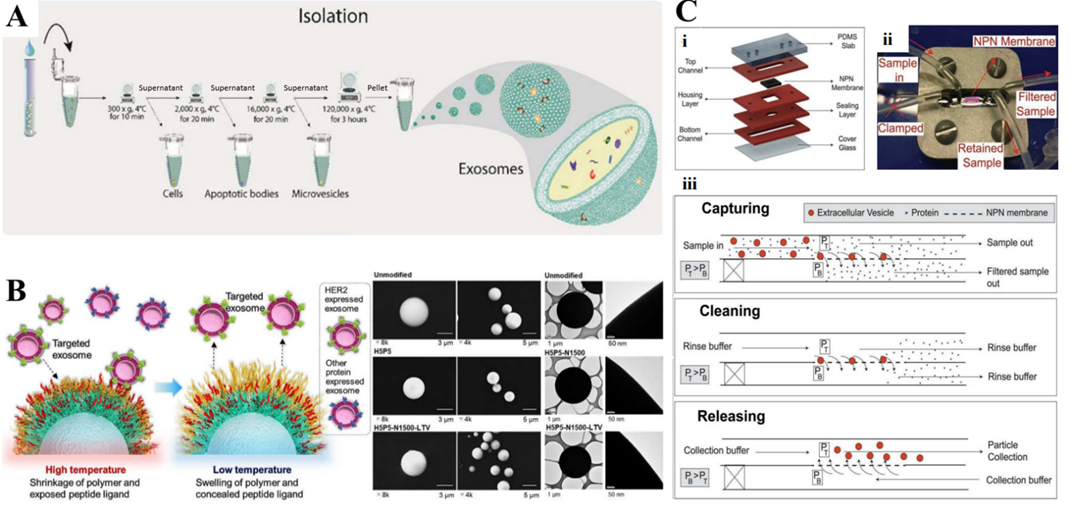
cells and other vesicles (>200 nm). Reproduced with permission. **[90]** Copyright 2022, Springer Nature. B) Affinity capture is achieved by designing corresponding antibodies against specific proteins present on the exosome surface. Reproduced under terms of the CC‐BY license. **[100]** Copyright 2023, Elsevier Ltd. C) Isolation of exosomes by tangential flow microfluidics: i) Assembly of the microfluidic device by a layer stacking process, where the channels are integrated into the sheet. ii) These sandwich layers are made monolithic by thermo-bonding or stacking and clamping. iii) Specific steps of tangential flow microfluidic chip for exosome capture. Owing to its integrated nature, the microfluidic chip can be used to isolate exosomes without being bound to a particular method, while combining multiple principles by means of multi-level separation. Reproduced under terms of the CC‐BY license. **[116]** Copyright 2022, Multidisciplinary Digital Publishing Institute.


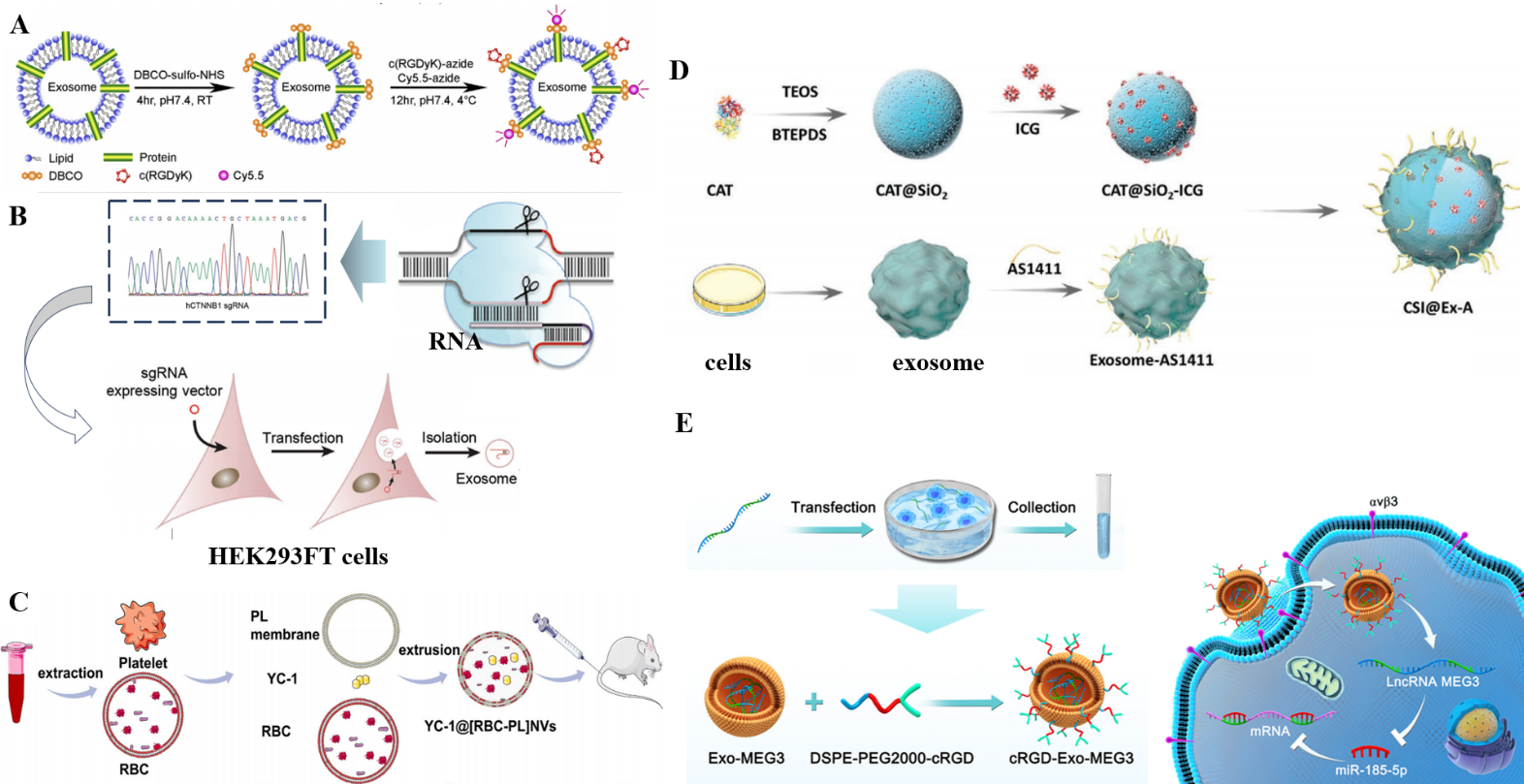
**Fig S3. Exosome loading drug modalities** A) Schematic representation of loading a drug using chemical modification: The α group (yellow) is first introduced to the surface of the exosome and forms a stable covalent bond with the protein (green) on it; subsequently, using click chemistry, the therapeutically effective *β* group (red) is combined with the α group, and the exosome is surface modified. Reproduced with permission. **[162]** Copyright 2018, Elsevier Ltd. B) Genetic engineering is utilized to control cellular protein expression, which in turn alters exosome content composition. Reproduced under terms of the CC‐BY license.**[164]** Copyright 2018, Wiley‐VCH. C) Schematic of a functionalized modification of exosomes using membrane fusion: Hybridized membrane nanovesicles composed of exosomes and platelet (PL) membranes encapsulate bioactive substances (yellow) for highly targeted delivery. Reproduced with permission. **[166]** Copyright 2023, Elsevier Ltd. D) A modification method that combines both surface modification and membrane fusion strategies is used. Reproduced with permission. **[184]** Copyright 2022, John Wiley and Sons. E) Exosome modification method combining both surface modification and genetic engineering. Reproduced with permission. **[185]** Copyright 2022, Elsevier Ltd.

**Table S1.** Membrane Fusion Technologies and Their Evaluation

| Method | Efficiency | Product Homogeneity | Effects on Exosome Integrity | Operational complexity | Scalability potential |
| --- | --- | --- | --- | --- | --- |
| Co-incubation | Low. The membrane fusion process is protracted, and since most exosomes and liposomes carry negative charges, electrostatic repulsion hinders spontaneous fusion. | Poor. The spontaneous fusion process is difficult to control and susceptible to environmental factors, potentially resulting in products of inconsistent size and composition. | Fairly good. No external damage occurs, allowing the original membrane integrity to be maintained reasonably well, but lipid-soluble drugs may escape during the process. | Simple. No special equipment required—just mix and incubate. Easy to operate. | Limited. Long reaction times, low efficiency, and potential product stability issues may restrict its large-scale application. |
| Co-extrusion | High. Fusion is forcibly promoted through mechanical force (extrusion through a membrane with specific pore size), significantly reducing the time required compared to co-incubation. | Good. Under external force, it readily forms vesicles of uniform size with good reproducibility. | Poor. Mechanical forces may cause partial rupture, inactivation, or deformation of exosomes/liposomes, with leakage of contents potentially contaminating the system. | Moderate. Requires the use of an extrusion device; the operating steps are relatively simple and controllable. | High. With few variables and excellent repeatability, it is considered one of the methods with the greatest potential for large-scale production. |
| ultrasound | High. Ultrasonic energy disrupts membrane structures, promotes fragment reorganization, and achieves rapid fusion rates. | Poor. After membrane rupture, reassembly occurs randomly, resulting in hybrid exosomes of varying sizes and poor homogeneity. | Poor. It completely disrupts the integrity of the original membrane, falling under the “destroy-rebuild” model. | Moderate. Requires ultrasonic equipment, with operation necessitating control of parameters ，such as time and power. | Moderate. The method itself is relatively straightforward, but the product is non-uniform, requiring additional purification steps that may increase complexity during scale-up. |
| freeze-thaw | High. Rapid fusion rate achieved by disrupting and reorganizing the membrane through freeze-thaw cycles (ice crystal formation and melting). | Poor. Similar to the ultrasonic method, the recombinant process is random, resulting in a broad product size distribution and poor homogeneity. | Poor. It completely disrupts the integrity of the original membrane, falling under the “destruction-reconstruction” model. | Simple. Only requires freezing and thawing equipment, with straightforward steps. | Moderate. The process is straightforward to operate, but it also faces issues such as product inconsistency and the need for subsequent purification. Additionally, there are certain limitations on the suitability of raw materials |
| Potential-guided membrane fusion | High. By inducing charge inversion—such as modifying one membrane to be positively charged—opposite charges attract each other, enabling spontaneous and efficient fusion. | Good. Under electrostatic forces, spontaneous ordered association promotes the formation of a homogeneous product. | Good. Without the need for strong external forces to disrupt them, the original structure and content integrity of the exosomes can be well preserved. | High. Chemical modification of the membrane is required to alter its potential, demanding advanced technical expertise and involving relatively complex procedures. | Promising potential. The fusion process is highly efficient and yields high-quality products, but the complex charge modification steps in the initial stages may become a bottleneck for large-scale production. While the technology is not yet mature, it holds significant development potential for the future. |
