# Supplementary figures and images for: Exosome Augmentation Technologies for Drug Delivery and Disease Treatment: A Review
Source: Biomater Res. 2026 Feb 19;30:0318. doi: 10.34133/bmr.0318 (PMC12917129; doi:10.34133/bmr.0318)

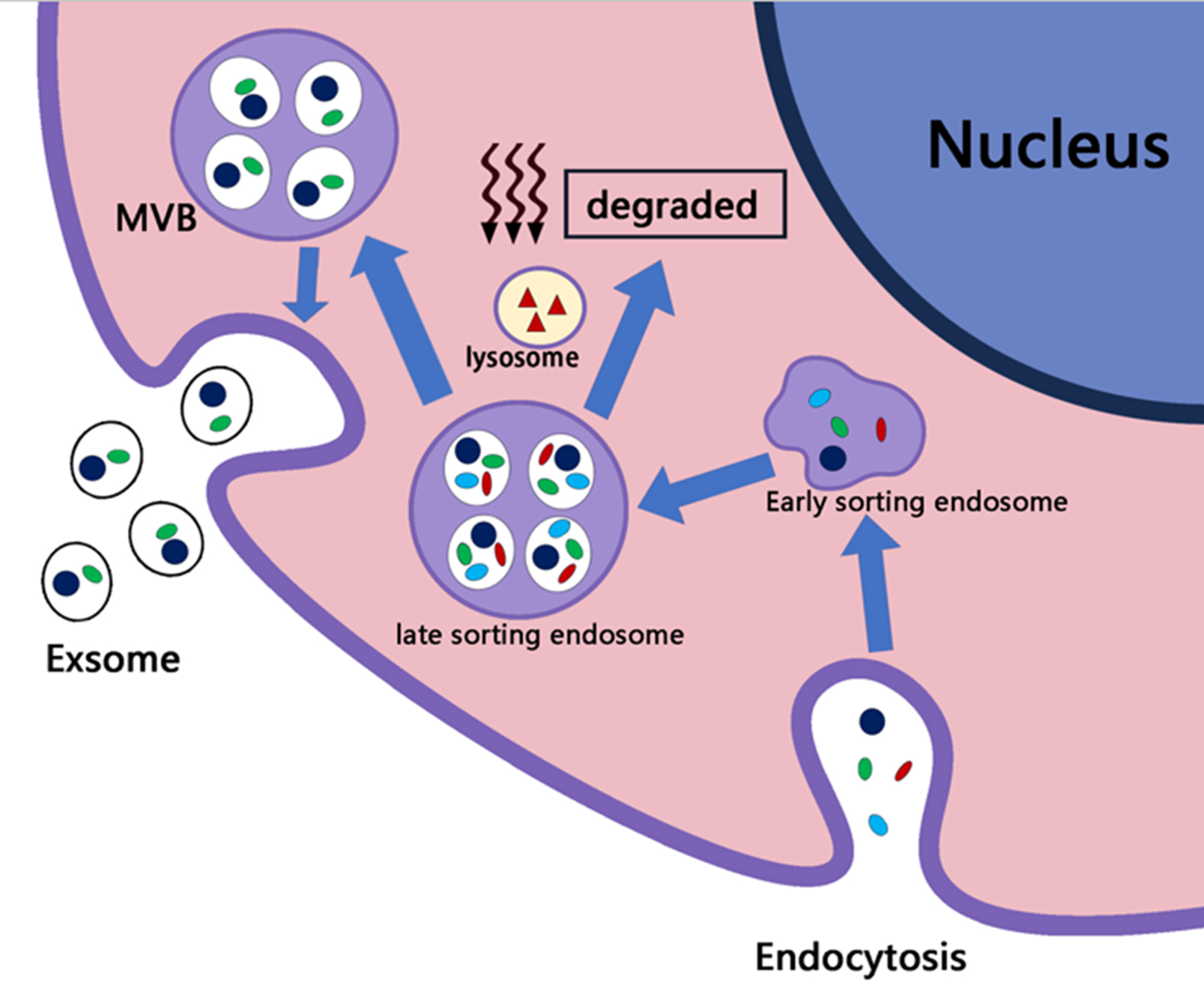

Supplement: Supplementary 1 — Figs. S1 to S3 Table S1 [file bmr.0318.f1.zip › support-Figure.1.png]

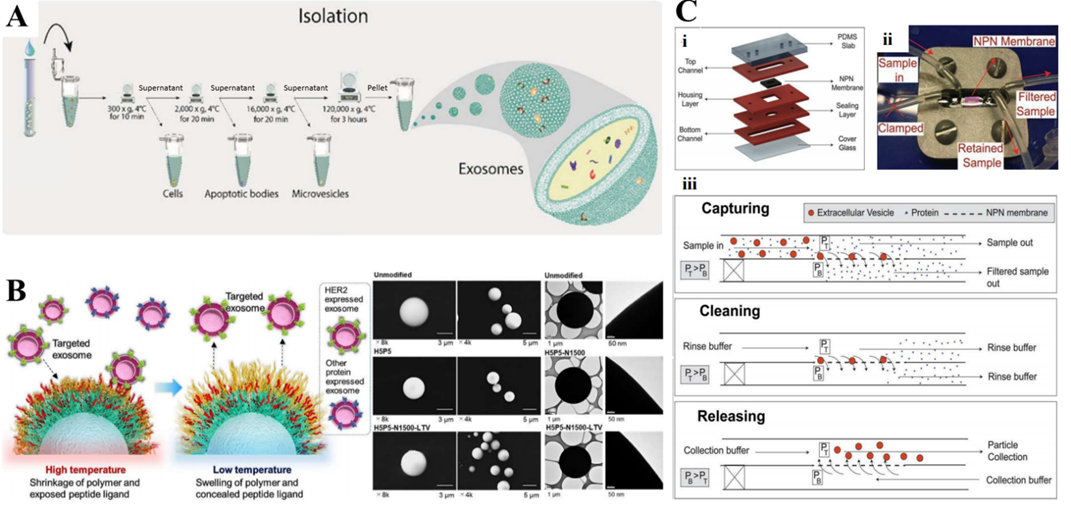

Supplement: Supplementary 1 — Figs. S1 to S3 Table S1 [file bmr.0318.f1.zip › support-Figure.2.png]

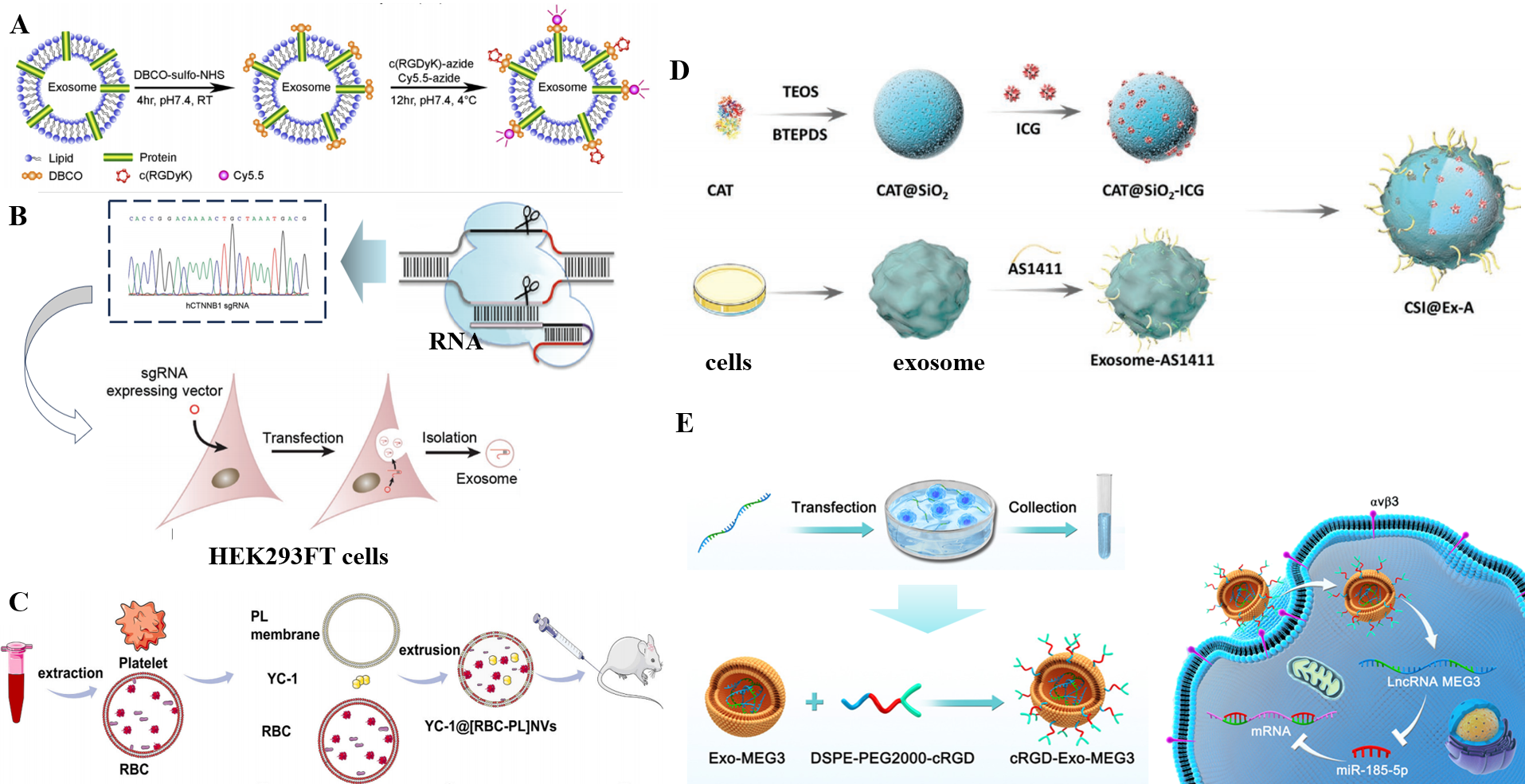

Supplement: Supplementary 1 — Figs. S1 to S3 Table S1 [file bmr.0318.f1.zip › support-Figure.3.png]
